# M1 macrophage-derived exosomes promote intervertebral disc degeneration by enhancing nucleus pulposus cell senescence through LCN2/NF-κB signaling axis

**DOI:** 10.1186/s12951-024-02556-8

**Published:** 2024-05-31

**Authors:** Chunyang Fan, Wei Wang, Zilin Yu, Jiale Wang, Wei Xu, Zhongwei Ji, Wei He, Di Hua, Wentao Wang, Linye Yao, Yongkang Deng, Dechun Geng, Xiexing Wu, Haiqing Mao

**Affiliations:** 1 Department of Orthopaedic Surgery, Orthopaedic Institute, The First Affiliated Hospital, Suzhou Medical College, Soochow University, Suzhou, Jiangsu China; 2 Department of Gastroenterology, The First Affiliated Hospital of Soochow University, Suzhou, China; 3 Department of Pain Management, Zhejiang Provincial People’s Hospital, People’s Hospital of Hangzhou Medical College, Hangzhou, Zhejiang China; 4 Department of Orthopaedic Surgery, Zhangjiagang Hospital Affiliated to Soochow University, Suzhou, Jiangsu China; 5 Department of Oncology, The First Affiliated Hospital of Soochow University, Suzhou, China

**Keywords:** Macrophage, Exosome, LCN2, Intervertebral disc degeneration, Cellular senescence

## Abstract

Intervertebral disc degeneration (IVDD) is the primary factor contributing to low back pain (LBP). Unlike elderly patients, many young IVDD patients usually have a history of trauma or long-term abnormal stress, which may lead to local inflammatory reaction causing by immune cells, and ultimately accelerates degeneration. Research has shown the significance of M1-type macrophages in IVDD; nevertheless, the precise mechanism and the route by which it influences the function of nucleus pulposus cell (NPC) remain unknown. Utilizing a rat acupuncture IVDD model and an NPC degeneration model induced by lipopolysaccharide (LPS), we investigated the function of M1 macrophage-derived exosomes (M1-Exos) in IVDD both in vivo and in vitro in this study. We found that M1-Exos enhanced LPS-induced NPC senescence, increased the number of SA-β-gal-positive cells, blocked the cell cycle, and promoted the activation of P21 and P53. M1-Exos derived from supernatant pretreated with the exosome inhibitor GW4869 reversed this result in vivo and in vitro. RNA-seq showed that Lipocalin2 (LCN2) was enriched in M1-Exos and targeted the NF-κB pathway. The quantity of SA-β-gal-positive cells was significantly reduced with the inhibition of LCN2, and the expression of P21 and P53 in NPCs was decreased. The same results were obtained in the acupuncture-induced IVDD model. In addition, inhibition of LCN2 promotes the expression of type II collagen (Col-2) and inhibits the expression of matrix metalloproteinase 13 (MMP13), thereby restoring the equilibrium of metabolism inside the extracellular matrix (ECM) in vitro and in vivo. In addition, the NF-κB pathway is crucial for regulating M1-Exo-mediated NPC senescence. After the addition of M1-Exos to LPS-treated NPCs, p-p65 activity was significantly activated, while si-LCN2 treatment significantly inhibited p-p65 activity. Therefore, this paper demonstrates that M1 macrophage-derived exosomes have the ability to deliver LCN2, which activates the NF-κB signaling pathway, and exacerbates IVDD by accelerating NPC senescence. This may shed new light on the mechanism of IVDD and bring a fresh approach to IVDD therapy.

**Graphical Abstract:**

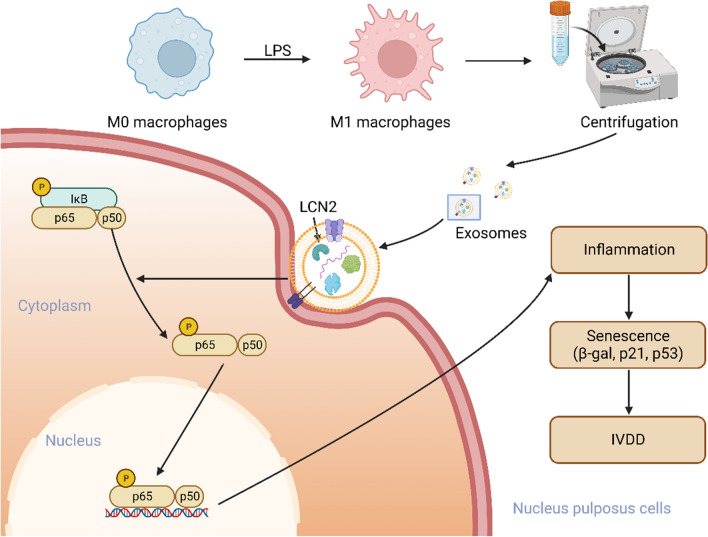

**Supplementary Information:**

The online version contains supplementary material available at 10.1186/s12951-024-02556-8.

## Introduction

Low back pain (LBP) is the leading cause of labor force loss and activity impairment in humans worldwide [1]. A total of 637 million people around the world suffer from LBP, causing enormous costs to household economies and national finances [2]. Intervertebral disc degeneration (IVDD) is a key factor leading to LBP [3]. However, there is no effective treatment for IVDD, which can only be achieved through medication or surgery to alleviate the patient's symptoms. Therefore, it is of great clinical significance to further study the pathogenesis of IVDD and to explore potential therapeutic targets in its development.

It is estimated that approximately 20% of adolescents have mild disc degeneration and 80% have LBP at some point in their lives, which is the biggest limiting factor for activity in people under 45 years of age [4]. Unlike in elderly individuals, in which lumbar degeneration is mainly caused by aging, injury or abnormal stress is often the cause of lumbar disc herniation in young people [5, 6]. In addition, Lee et al. found that the injury also aggravated disc herniation [7]. Damaged intervertebral discs (IVDs) can lead to changes in the microenvironment, leading to a series of pathological reactions, such as proinflammatory and catabolic reactions, tissue defects and biomechanical changes [8]. Disturbance of the IVD microenvironment attracts inflammatory cells to the IVD, which in turn stimulates resident IVD cells to produce additional proinflammatory cytokines [9, 10]. Therefore, it makes sense to better understand the properties and functions of these inflammatory cells to open up new therapies for IVDD.

Nerlich et al. discovered that the degraded IVD included inflammatory cells that expressed the panmacrophage marker CD68, revealing the key role of macrophage in the pathophysiology of IVDD [11]. Macrophages are predominant immune cells that regulate the onset, course, and resolution of inflammation. The M1 and M2 phenotypes, which are classically and alternatively activated, respectively, are involved in a range of acute and chronic illnesses [12]. Immuno-inflammation, especially M1 macrophage regulation, has a significant regulatory function during IVDD. In response to acute injury, M1 macrophages promote a high level of proinflammatory cytokine production (TNF-α, IL-1β, IL-6, IFN-γ), chemokines, and matrix metalloproteinases, thereby accelerating IVDD [13]. Dou et al. found melatonin could inhibit M1 macrophage polarization by modulating the SIRT1/Notch signaling pathway to improve NPC damage caused by inflammation [14]. By blocking the HMGB1/Myd88/NF-κB pathway and the NLRP3 inflammatome, Magnoliine mitigated the IVDD caused by M1 polarized macrophages [15]. Therefore, M1 macrophages may be crucial to the IVDD process, but the exact mechanism is still unknown, so in-depth investigation of its mechanism may provide a fresh perspective on the occurrence and development of IVDD.

Exosomes are bilayer membranous vesicles that range in diameter from 40 to 150 nm that are present in almost all biological fluids and contain a variety of substances, including cytokines, proteins, lipids, and various RNAs [16, 17]. Exosomes have a special role in mediating information transmission between cells according to the origin of the cells. For example, the COVID-19 cytokine storm was weakened by delivering dexamethasone using exosomes [18]. One major source of exosomes is macrophages. The biomolecular composition of exosomes released by macrophages reflects a special feature of the original cell: robust regulation of inflammation or immunity [19]. According to Jiang et al., via upregulating the TLR5/NF-κB pathway, lncRNA HOTTIP in exosomes produced from M1 macrophages suppressed the advancement of head and neck squamous cell cancer [20]. Liu et al. found that in the myocardial infarction microenvironment, exosomes generated from M1-like macrophages transmit miR-155, which exacerbates heart dysfunction and inhibits angiogenesis [21]. Sharma found that long-term intake of high-fat diet could polarize monocytes into M1 macrophages in adipose tissue, produced CCL2 and TNF-α, accelerated inflammatory response, and led to the occurrence of metabolic syndrome [22]. In addition, M1-Exos acted as a pro-inflammatory mediator, activated TLR4 signaling, destroyed the mucosal barrier, and accelerated the progression of colitis in mice [23]. M1-Exos enhanced anti-tumor activity of paclitaxel by activating macrophage-mediated inflammatory response [24]. A multitude of disorders are impacted by exosomes produced from M1 macrophages. For this reason, we postulated that exosomes secreted by M1 macrophages may be crucial in IVDD.

A conserved cell fate throughout evolution, cellular senescence is characterized by stable replication stagnation brought on by various stresses [25]. It is characterized by increased expression of cell cycle regulators p16^INK4A^ and p21^Cip1^, increased expression of SASP factors (such as IL-6 and IL-8) and increased β-galactosidase activity [26]. Cellular senescence is an important cause of IVDD, which is closely related to the enhancement of inflammatory response [27]. SIRT3 can delay oxidative stress and inflammatory response, thereby delaying the senescence of NPCs to reduce IVDD [28]. Similarly, activation of CB2R can inhibit the inflammatory response, delay the aging of NPCs, restore the balance of extracellular matrix metabolism, and alleviate IVDD [29]. Inhibition of inflammatory response can delay the senescence of nucleus pulposus cells. Yu et al. found that M1 macrophages promoted ROS accumulation, thereby enhancing glomerular endothelial cell senescence [30]. Therefore, we speculate that M1 macrophage derived exosomes can play a proinflammatory role, promote cellular senescence, and then aggravate IVDD.

Our hypothesis in this work was that exosomes generated from M1 macrophages enter the NPCs to play a proinflammatory role and affect the pathological process of IVDD. We used an LPS-induced degeneration model of NPCs and a rat acupuncture model to investigate the effect of M1-Exos on IVDD. Furthermore, the exosome inhibitor GW4869 was used to intervene in vivo and in vitro. We found that M1-Exos can accelerate the senescence of NPCs, disrupt ECM metabolism, and ultimately aggravate IVDD. We investigate potential mechanisms to improve our understanding of IVDD and provide a new perspective and frame of reference for subsequent research.

## Materials and methods

### Animals

A total of fifty three-month-old male Sprague‒Dawley rats (n = 50) with a weight of 450 ± 50 g were chosen from the Soochow University Animal Center. In a light/dark cycle lasting 12/12 h at 21 °C, the rats were housed.

### Clinical study

The First Affiliated Hospital of Soochow University's Ethics Review Committee gave its approval for the clinical investigation. Consent was granted in writing to each participant.

A total of 727 patients with lumbar disc herniation (LDH) who were admitted to our department between September 2022 and September 2023 were examined retrospectively in this research. Seven age categories were used to count the population. Sixty patients were chosen at random and split evenly into two groups: the young group (≤ 45) and the old group (≥ 45). MRI and cytokine levels were also recorded.

### Preliminary processing of single-cell sequencing data

The single-cell sequencing data, which included the sequencing data of eight disc degeneration tissues, were obtained from the GEO database (registration number GSE165722). Single-cell data processing often uses the Seurat program (version 4.1.1). Quality control is based on the following criteria: (1) Take out any cells that have more than 6000 genes or less than 500 genes; (2) Every cell sequenced must have a UMI count value larger than 1000, and the top 3% of cells with the highest UMI count value must be disregarded; (3) Every cell’s percentage of expressed mitochondrial genes should not exceed 35% of all genes, and the top 2% of cells exhibiting the greatest percentage of expressed mitochondrial genes should be eliminated; (4) Determine the ratio of rRNA expression to total gene expression and exclude the top 1% of cells with the lowest and top 1% of cells with the highest proportions, respectively. The harmony technique, which is based on the harmony package (version 0.1.0), was used to integrate and debatch 8 samples to prevent batch effects on downstream analysis. We solve this problem by normalizing counting data to generate similar relative gene expression abundances across cells using the normalization function, which helps to avoid mistakes in downstream analysis caused by technical discrepancies.

### Dimension reduction and visualization of the single-cell sequencing matrix

Using the FindVariableFeatures tool, we selected 2000 high-variability genes–genes that contribute information to the data’s variability—as features. The z score values of gene expression were converted to conform to the Gaussian distribution using the ScaleData function. Subsequently, we employed the PCA algorithm’s linear dimensionality reduction technique to map the expression matrix into a low-dimensional space while simultaneously capturing all of the data's information in as few dimensions as possible. This allowed us to determine the lowest dimension at which the biological form formed by the cell expression map could be displayed. Once the linear size reduction is finished, we identify the appropriate dimensions and apply the UMAP approach to the data to complete the nonlinear dimensionality reduction, which allows us to ultimately map the multidimensional data to the two dimensions that are appropriate for observation. Finally, we use the FindNeighbors function to build a KNN plot based on Euclianian distances in a PCA space and refine the edge weights between any two elements based on shared overlap (Jaccard similarity) in their local neighborhood. For the optimization of conventional modular functionality (resolution = 0.5), cell iterations are clustered using the FindClusters function.

### Annotation and reclassification of cell subpopulations

We have synthesized many annotation approaches to achieve the highest accuracy in cell subset annotation. Using the singleR package (version 1.8.1), we start with a basic annotation of individual cell subpopulations. Next, we searched for genes that were differentially expressed between each subpopulation and every other subpopulation (filter threshold P < 0.05) using the FindAllMarkers tool. We manually annotated each cell subpopulation one by one after obtaining the cell marker genes. Our annotation was based on a thorough review of the literature and internet resources such as CellMarker and BMC Genome Biology. We then extracted all macrophage subpopulations and reclassified them. Once again, normalization, centralization, dimensionality reduction in PCA and UMAP, identification of cell subgroups, and discovery of highly variable genes are the procedures involved in reclassification. Finally, using M1 and M2 macrophage surface markers as a basis, we further identified the macrophage subgroups.

### Preparation of M0 and M1-Exos from cell medium

In a 15-cm culture plate, RAW264.7 cells were inoculated to produce M1 exosomes. LPS (100 ng/mL) induced M1-polarized macrophages when the cell confluence reached 70%. Then, as previously mentioned, M0 and M1-Exos were separated from the conditioned medium [31]. The debris was first disposed of by centrifugation at 2000*g* (15 min) and 10,000*g* (30 min) at 4 °C and then filtered through a 0.22 μm filter (Millipore). Second, using a superfast centrifuge (Himac), the medium was spun for two hours at 120,000*g*. Thereafter, PBS was used to resuspend the M0 and M1-Exo particles.

GW4869 was initially dissolved in DMSO. To inhibit exosome production, RAW264.7 cells were pretreated with 3 μM GW4869 for 24 h before LPS treatment. Exosomes were extracted by collecting supernatant.

### Characterization of M0 and M1-Exos

M0 and M1-Exo concentrations were examined to identify the existence of their markers using the Micro BCA Protein Assay kit (Thermo, USA), and TSG101 and CD63 were detected by western blot. Particle concentration and quantity-weight diameter were estimated using nanoparticle tracking analysis (NTA, Malvern, UK). Transmission electron microscopy (TEM, HITACHI) was used to examine and study the morphology of M0 and M1-Exos. First, 150-mesh carbon-coated copper grids were pipetted with one drop of M0 or M1-Exos (20 μL) (Servicebio). After using filter paper for a minute to dry the extra liquid, the sample was colored for a minute using 2% phosphotungstic acid (Zcibio). After being fluorescently dried for ten minutes, the samples were examined by TEM.

### Chemicals and antibodies

Chemicals, primers, antibodies, siRNAs, and plasmids are all listed in detail in Table S1.

### Rat NPC culture

Six-week-old Sprague‒Dawley (SD) rats were killed with a single intraperitoneal injection of excess pentobarbital (500 mg/kg). Next, the NP tissue from the rat caudal intervertebral disc was separated and processed for two hours at 37 °C in a water bath using 0.5% type II collagenase. Upon centrifugation, the precipitate was collected and reconstituted in a 6-well plate containing 15% fetal bovine serum (FBS; Invitrogen, Waltham, MA, USA) and 1% penicillin/streptomycin in Dulbecco’s modified Eagle’s medium/nutrient mixture (DMEM/F12; Invitrogen). The cell plates were kept at a consistent 37 °C in an incubator with 5% CO_2_. The cells were digested with 0.25% trypsin–EDTA (Invitrogen) when reaching 70–80% of the total cell density. We employed cells that were no older than three generations in our experiment.

### RNA interference

Gene Pharma provides siRNA (RNA oligonucleotides). The 6-well plate was supplemented with diluted siRNA/plasmid and GP transfection partner after the NPC attained 60–70% satiation. The dish was then grown in serum-free media. The complete medium was applied to each well after four hours. The degree of LCN2 protein expression served as a proxy for the transfection effect.

### SA-β-gal staining

An appropriate amount of SA-β-gal staining fixative was cultured after the intervention and left to fix at room temperature. A suitable quantity of staining solution (Beyotime) was obtained, cleaned with PBS, applied to the culture wells, and kept at 37 °C without the presence of CO_2_ for 24 h. The last step was to use a high-resolution microscope to capture pictures.

### Real-time quantitative polymerase chain reaction (RT-qPCR)

Following a normal procedure, RNA was extracted from NPCs. Using a NanoDrop 2000 spectrophotometer (Thermo Fisher Scientific, Waltham, MA, USA), the concentration of RNA was measured. Afterwards, the RNA/reverse transcriptase mixture was used to create cDNA. Forward and reverse primers and qPCR MasterMix (Biotium) were used for the PCR amplification process. Using the 2^−ΔΔCq^ method, the mRNA expression data were analysed. All primers were provided by Shenggong Biotech (Shanghai, China). Table S2 displays the exact primers.

### Western blot analysis

The concentration of the total protein was determined using the BCA protein assay kit (Beyotime), which was produced by dividing NPCs in a six-well plate using radioimmunoprecipitation (RIPA) buffer (NCM Biotech, Soochow, China). A polyvinylidene fluoride membrane (Bio-Rad Laboratories) was used to transfer an equivalent quantity of protein (10 μg) that had been obtained using sodium dodecyl sulfate‒polyacrylamide gel electrophoresis. Following one hour of blocking at room temperature in Beyotime's QuickBlock block solution, the membrane was subjected to primary and matching secondary antibody treatments. The separated bands were observed by chemiluminescence (Pierce ECL). By using optical density analysis, the autoemission map was examined. The program Image Lab 2.1 was used to calculate the relative gray levels.

### Surgical procedures

After the rats were given abdominal anesthesia, use fingers to roughly determine the target caudal vertebral segment, place a metal marker, and confirm under X-ray. The 21G puncture needle was placed perpendicular to the skin, parallel to the vertebral endplate, and inserted into the median of the intervertebral space at a depth of about 10 mm for 30 s. X-ray was performed again to confirm that the tip of the needle was located in the target space and in the center of the intervertebral space at both the anterior and lateral positions. After puncture, GW4869 (2 mg/kg) was injected into the corresponding caudal disc. Imaging tests were performed 7 days later.

### X-ray and magnetic resonance imaging (MRI) analysis

One week after surgery, MRI and X-ray were performed on each rat's tail before death. The rats were placed in a supine posture, and an X-ray machine made by General Electric Company (USA) was used to examine their tails. The parameters were as follows: exposure of 63 MA, penetration of 35 kV, and collimator distance of 66 cm from the film. A 1.5-T system (GE, USA; repetition time 3000 ms, echo time 80 ms, field of view 200 mm^2^, scanning thickness 1.4 mm) was used to acquire MRI T2-weighted images, which were then processed using imaging software (DICOM 3.0, Neusoft PACS/RIS). The IVD height index of the rats was then determined by measuring and computing it using software. ImageJ software 2.1 (Bethesda, MD, USA) was used to assess the optical density of IVD.

### Hematoxylin and eosin staining (H&E) and safranin-O staining

For the purpose of decalcification, rat tail vertebra specimens were submerged in 10% formalin for 48 h and 10% EDTA for 30 days. After that, paraffin was embedded in the fixed sample. On slices that were 5 µm thick, H&E and safranin-O staining were applied. Masuda assessed the histological grade after examining samples under a microscope.

### Paraffin specimen immunofluorescence staining

Paraffin slices were dewaxed, hydrated, and then sealed for 30 min at room temperature using 10% goat serum. A primary antibody (1:200) was added, and the sample was incubated overnight at 4 °C. The paraffin slices were stained with DAPI for ten minutes on the second day after being treated for thirty minutes with the corresponding fluorescent secondary antibody (Alexa Fluor^®^647, Abcam).

### Immunocytochemical (ICC) staining

NPCs were treated with several intervention techniques and plated at a density of 10,000/ml in a 24-well plate. The cells were treated with PBS washing, 4% paraformaldehyde fixation, and Triton infiltration after the intervention. The cells were exposed to the primary antibody overnight after the nonspecific binding sites were blocked using a fast blocking solution. They underwent a one-hour incubation period with the secondary antibody and a ten-minute DAPI staining process the next day.

### Statistical analysis

GraphPad Prism 8 (GraphPad Software Inc., La Jolla, CA) was used to process the data, and the results are shown as the mean ± standard deviation (SD). Student’s t test was used to compare and evaluate the data from the two groups. One-way ANOVA was used to compare data among multiple groups. A significant difference was defined at *P* < 0.05, and a highly significant difference was defined at *P* < 0.01. (*P < 0.05, **P < 0.01).

## Results

### Inflammatory infiltration is an important factor in IVDD in young patients

First, we found that among 727 LDH patients in our hospital, 50–70 years old was the highest incidence age, followed by 30–39 years old (Table 1). Statistically, 29% of LDH patients were younger than or equal to 45 years old, while 71% of LDH patients were older than 45 years old (Fig. S1A). Then, according to Zhou et al.’s research [32], we split the sixty LDH patients into two age groups based on their age: the young group and the old group. MRI showed that in young patients, the IVD signal was still good in all segments except the degenerative IVD, and a high-intensity zone (HIZ) was visible in the degenerative disc, while in elderly patients, all segments of the disc were significantly degraded, and the overall stability of the lumbar spine was also worse (Fig. S1B). We further analysed the cytokines and found that the levels of IL-6, IL-1β and IL-17A in the young group were significantly higher than those in the old group. There was no significant difference between IL-4 and IL-10. Unexpectedly, there was no discernible difference in TNF-α levels between the two groups (Table 2). Therefore, we speculate that IVDD in young patients may be related to inflammation.

**Table 1:** Number of patients with lumbar disc herniation

Age	Number	Rate (%)
< 20	5	1
20–29	33	4
30–39	115	16
40–49	103	14
50–59	159	22
60–70	199	2
> 70	113	16

**Table 2:** The results of cytokines

	Young (n = 30)	Old (n = 30)	*P* value
Age(years)	33.60 ± 6.75	66.40 ± 5.50	< 0.0001
IL-6			< 0.0001
Abnormal	18	4	
Normal	12	26	
IL-1β			0.035
Abnormal	16	8	
Normal	14	22	
IL-17A			< 0.0001
Abnormal	20	5	
Normal	10	25	
TNF-α			0.067
Abnormal	16	9	
Normal	14	21	
IL-10			0.448
Abnormal	3	5	
Normal	27	25	
IL-4			0.796
Abnormal	14	15	
Normal	16	15	

### M1-dominated macrophage infiltration was present in IVDD

**Fig. 1:**
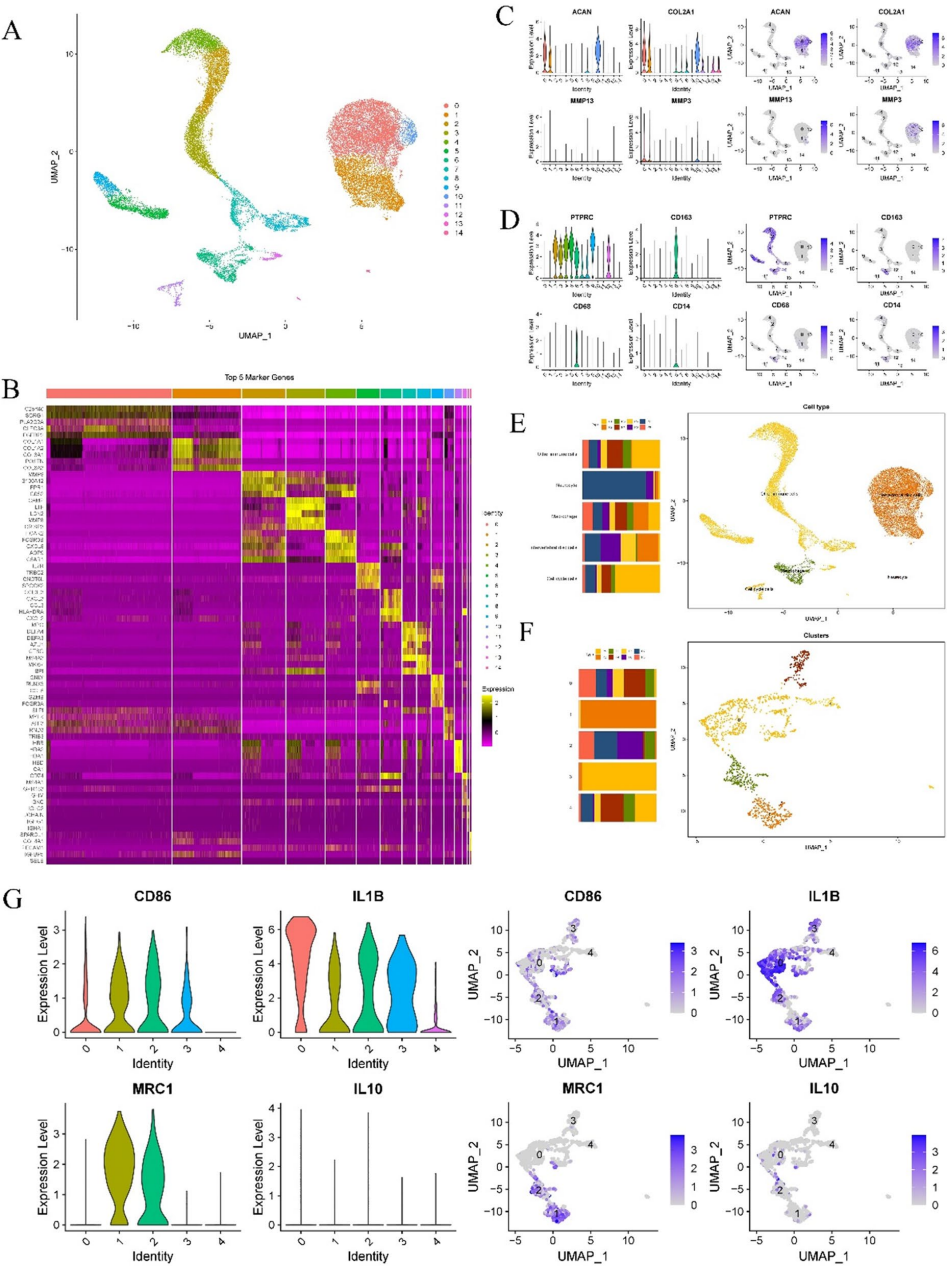
M1-dominated macrophage infiltration was present in IVDD. **A** Single cell expression matrix reduction of 8 degenerated intervertebral disc tissues. **B** Heat maps of specific high-expression genes in the top 5 of 15 cell subsets. **C** Relative expression levels of intervertebral disc cell markers in single cell expression matrix. **D** The relative expression levels of immune cell markers and macrophage markers in single cell expression matrix. **E** Final annotation results of single cell expression matrix. **F** UMAP dimension reduction results of macrophage reclassification.** G** The relative expression levels of M1 and M2 macrophage markers in the macrophage expression matrix

Based on the above treatment methods, we obtained the single-cell expression matrix of disc degeneration tissue. The single-cell expression matrix consisted of 15 cell subsets (Fig. 1A). We annotated the cell subpopulations by identifying the specific highly expressed genes of each cell subpopulation. The heatmap shows the top 5 most specific genes in each cell subpopulation (Fig. 1B). Based on previous studies, we identified IVD cell markers. The IVD cells (subgroups 0, 1, 10) were annotated by the ACAN, COL2A1, MMP13, and MMP3 genes (Fig. 1C). In addition, we found that subpopulations 2, 3, 4, 5, 6, 7, 8, 9, and 12 were immune cells. According to the macrophage markers CD163, CD68 and CD14, subgroup 8 was further identified as macrophages (Fig. 1D). The remaining cell subsets were also annotated according to the corresponding highly expressed genes, such as subgroup 11 for cell cycle cells and subgroup 14 for nerve cells. Finally, we obtained annotated results for all cell subpopulations (Fig. 1E). Subsequently, macrophages (subgroup 8) were extracted for reclassification, and the results showed that infiltrated macrophages in the disc degenerative tissue were divided into 5 subgroups (Fig. 1F). Among them, subgroups 0, 1, 2 and 3 highly expressed the M1 macrophage markers CD86 and IL1B, while subgroups 1 and 2 also expressed the M2 macrophage marker MRC1, and IL10 was almost not expressed in any cell subgroup (Fig. 1G). Therefore, we determined that subpopulations 0 and 3 were M1 macrophages, and subpopulations 1 and 2 were mixed cell populations of M1 macrophages and M2 macrophages. This suggests the presence of M1-dominated macrophages in the degenerated IVD.

Next, we verified this with tissue fluorescence staining. The findings demonstrated that compared to normal tissues, the degraded IVD tissues had much greater levels of CD86 expression (Fig. S2). This suggests that M1 macrophages, as important inflammatory cells, have extensive infiltration in degraded IVD tissue.

### Extraction and identification of M1 macrophage-derived exosomes and their effects on IVDD

**Fig. 2:**
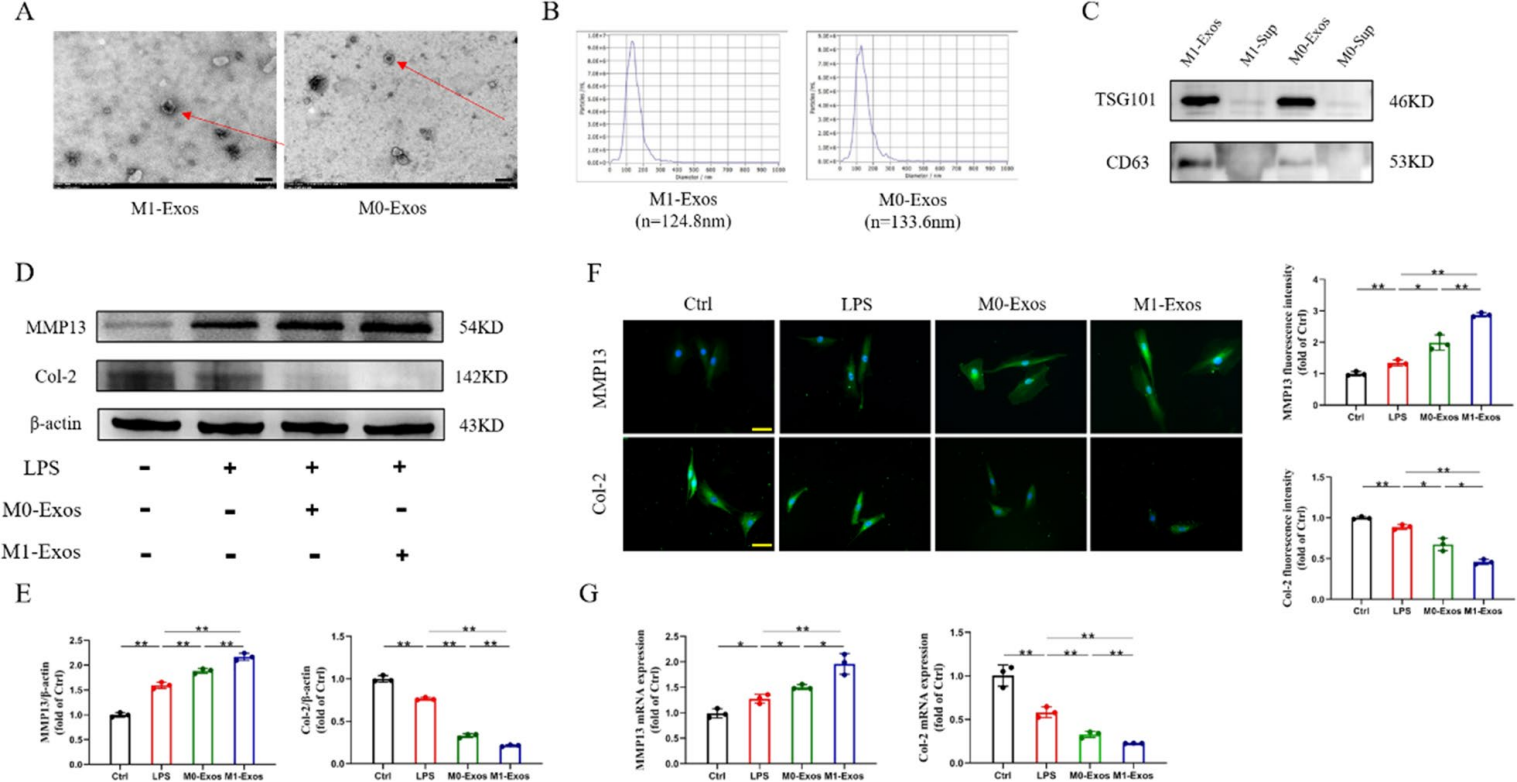
Extraction and identification of M1 macrophage-derived exosomes and their effects on IVDD. **A** Representative TEM images of M1-Exos and M0-Exos. Scale bars, 200 nm.** B** Representative chart of exosome size distribution measured by dynamic light scattering method. **C** WB analysis of Exos markers CD63 and TSG101 in the supernatant (Sup) and exosomes (Exos). **D**, **E** The expression and quantification of IVDD-specific proteins.** F** Representative fluorescence images of IVDD-specific markers. Scale bars, 50 μm. **G** The expression of IVDD-specific genes. ns: no significance, *P < 0.05, **P < 0.01

To extract M1 macrophage-derived exosomes, ultracentrifugation was used to obtain exosomes from the supernatant of M1 macrophages (Fig. S3). As shown in Fig. 2A, under TEM, both M0-Exo and M1-Exo showed spherical vesicles and bilayer lipid membranes. We used NTA to measure the size distribution of M0-Exos and M1-Exos with diameters of 133.6 nm and 124.6 nm, respectively, both between 40 and 150 nm (Fig. 2B). Subsequently, Western blotting showed that M0-Exos and M1-Exos expressed the exosome-specific proteins TSG101 and CD63 (Fig. 2C). These results showed that exosome extraction was successful.

Next, we explored the effect of M1-Exos on IVDD. Firstly, LPS (1 μg/ml) was used to induce NPC degeneration, and then 10 μl (about 10 μg) of M0-Exos and M1-Exos were added respectively for 24 h. The imbalance of ECM metabolism is the fundamental mechanism of IVDD, which is characterized by inhibiting the anabolism of NP matrix proteins (such as Col-2) and inducing the secretion of matrix metalloproteinases (such as MMP13)[33]. According to Western blot analysis, LPS increased the expression of MMP-13 while decreasing the expression of Col-2. These changes were exacerbated by M1-Exos (Fig. 2D, E). ICC staining showed a greater distribution of Col-2 around the nucleus in the Ctrl group than in the LPS group, while the expression and distribution decreased in the M1-Exo group. The opposite is true for MMP13 (Fig. 2F). The outcomes of RT‒PCR were identical (Fig. 2G). These results suggest that M1-Exos can aggravate ECM degradation in NPCs in IVDD.

### Targeted regulation of M1-Exos can affect NPC senescence

**Fig. 3:**
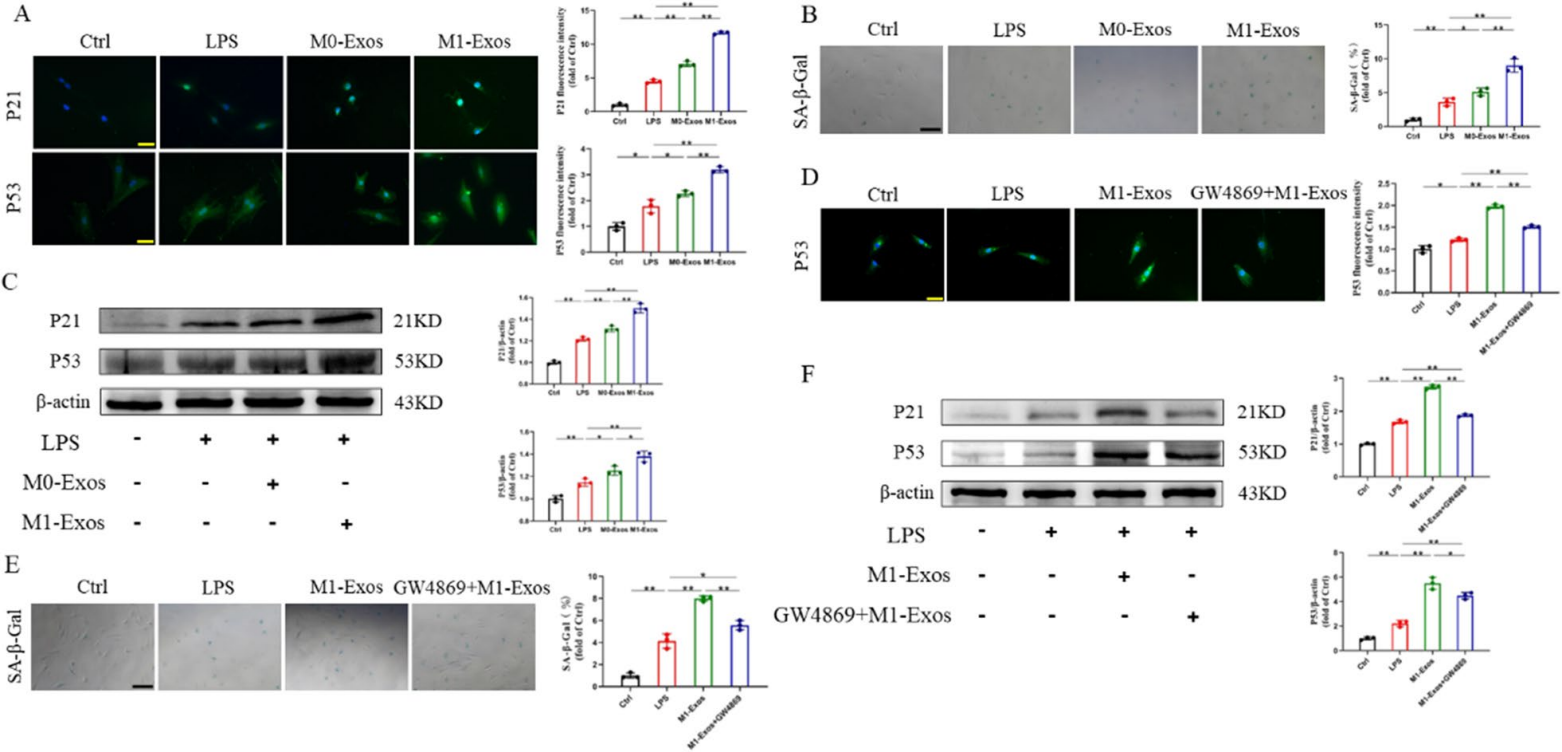
Targeted regulation of M1-Exos can affect the senescence of NPCs. **A** Representative fluorescence images of senescence markers after addition of M1-Exos. Scale bars, 50 μm. **B** SA-β-gal staining and quantitative analysis results after addition of M1-Exos. Scale bars, 100 μm. **C** The expression and quantification of senescence proteins after addition of M1-Exos. **D** Representative fluorescent images of senescence markers after GW4869 intervention. Scale bars, 50 μm. **E** SA-β-gal staining and quantitative analysis results after GW4869 intervention. Scale bars, 100 μm. **F** The expression and quantification of senescence proteins after GW4869 intervention. ns: no significance, *P < 0.05, **P < 0.01

Next, we further explored the role of M1-Exos in NPC senescence. According to immunofluorescence, the LPS group had considerably increased P53 expression compared to the Ctrl group; however, compared to the LPS group, P53 expression was increased by 27.4% in the M0-Exos group and 79.4% in the M1-Exos group. P21 had the same result (Fig. 3A). In addition, there were 3.6 times more SA-β-gal-positive cells in the LPS group than in the Ctrl group, and this result was exacerbated by M0-Exos and M1-Exos, which were 1.4 times and 2.5 times more than LPS, respectively (Fig. 3B). Western blot and RT‒PCR produced comparable findings (Fig. 3C, Fig. S4A).

To investigate the connection between M1-Exos and NPC senescence in more detail, we used the exosome inhibitor GW4869 to regulate the production of M1-Exos. According to Guo et al. [34], to inhibit the production of exosomes, macrophages were pretreated with GW4869 before LPS treatment, and the culture supernatant was collected for exosome isolation. As shown in Fig. 3D, E, P53 expression and the percentage of SA-β-gal-positive cells were considerably greater in the M1-Exos group than in the Ctrl and LPS groups, but pretreatment with GW4869 dramatically reduced both of these parameters. In addition, the mRNA and protein levels of P53 and P21 had very comparable patterns, which is in line with the SA-β-gal staining findings (Fig. 3F, Fig. S4B). In summary, M1-Exos can accelerate the senescence of NPCs, but this effect was blocked by treating macrophages with the exosome inhibitor GW4869, which significantly reduced the secretion of M1-Exos.

Finally, we further investigated the effect of M1-Exos on ECM degradation in NPCs (Fig. S5). Immunofluorescence staining showed that M1-Exos derived from supernatant pretreated with GW4869 partially reversed the decrease in Col-2 expression and the increase in MMP13 expression after M1-Exo intervention in NPCs. In addition, RT‒PCR and western blot results showed that the expression level of Col-2 in NPCs in the M1-Exos group was significantly lower than that in the LPS group under inflammation. In contrast, the expression of MMP13 in the M1-Exos group was significantly higher than that in the LPS group. At the same time, M1-Exos derived from supernatant pretreated with GW4869 saved the decrease in Col-2 expression and inhibited the increase in MMP13 expression induced by inflammation. This suggests that a reduction in the synthesis of M1-Exos can attenuate aggravated NPC degeneration.

### Inhibition of M1-Exos can delay the senescence of nucleus pulposus cells in vivo

**Fig. 4:**
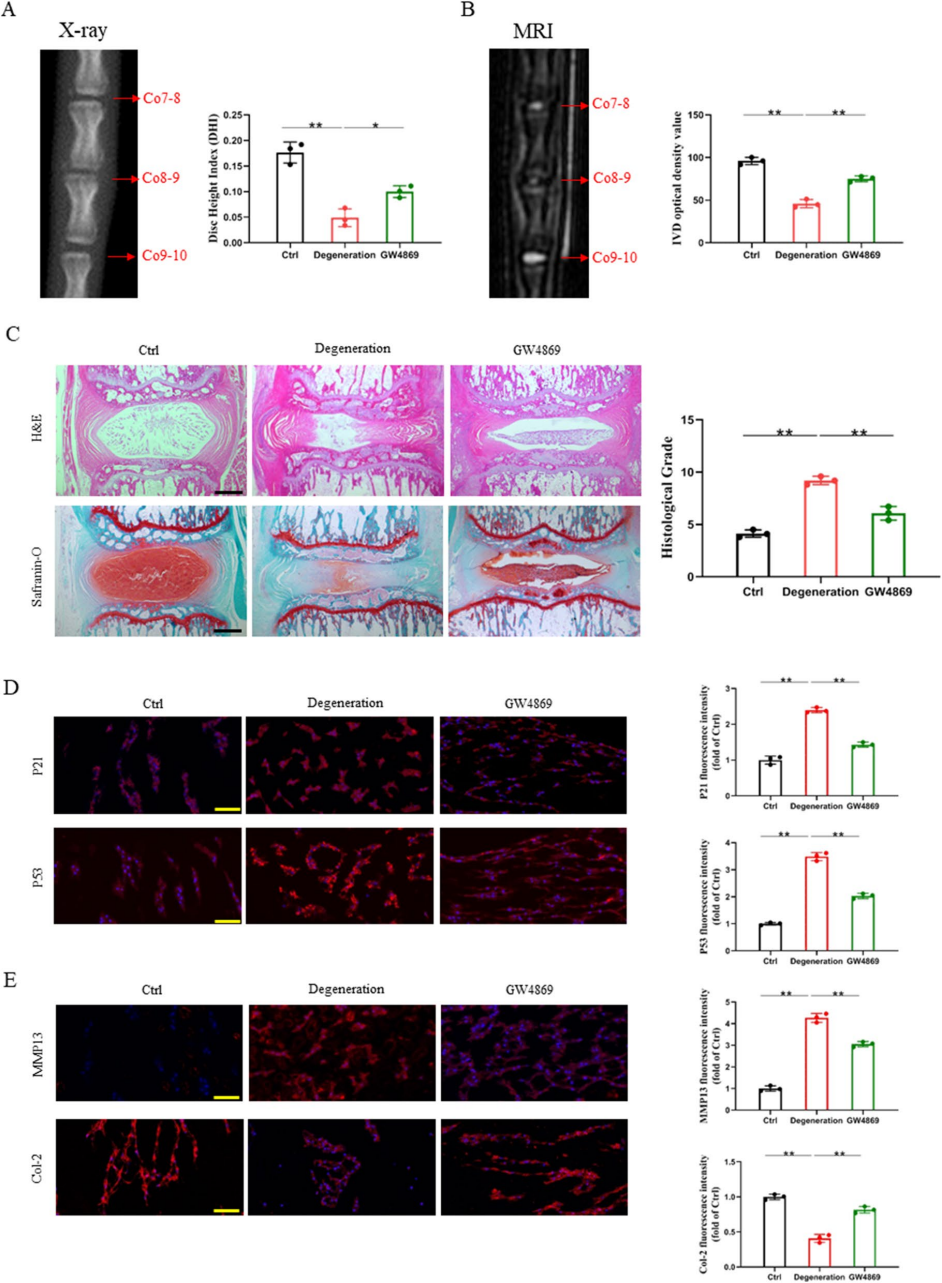
Inhibition of M1 macrophage-derived exosomes can delay the senescence of nucleus pulposus cells in vivo. **A** X-ray examination of rat caudal at 1 week after surgery. **B** T2-weighted MRI scans of rat caudal at 1 week after surgery. **C** H&E and safranin-O staining of rat caudal IVD and histological grade. Scale bars, 1000 μm. **D** Representative fluorescent images of senescence markers after GW4869 intervention. Scale bars, 50 μm. **E** Representative fluorescent images of IVDD-specific markers after GW4869 intervention. Scale bars, 50 μm. ns: no significance, *P < 0.05, **P < 0.01

In order to learn more about how M1-Exos affect IVDD in vivo, we established a rat acupuncture model and used the exosome inhibitor GW4869 to intervene. The X-ray results showed that the disc height index (DHI) in the degeneration group decreased by 51.0% compared with that in the Ctrl group. The DHI in the GW4869 group was 2.0 times higher than that in the degeneration group (Fig. 4A). The outcomes of the X-ray and MRI exams agreed with one another. IVD light intensity dropped by 52.1% in the degeneration group compared to the Ctrl group, according to quantitative MRI data. When comparing the GW4869 group to the degeneration group, there was a 63.2% rise in the IVD light intensity (Fig. 4B).

Furthermore, histological staining techniques such as safranin-O and H&E staining supported the conclusions drawn from the aforementioned imaging data. NP in the Ctrl group was found to be generally complete, having a large and full oval form. Nonetheless, acupuncture dramatically quickens NP tissue degeneration, which leads to the loss of NP tissue and the development of smaller, irregular shapes. However, the injection of GW4869 at the acupuncture site mitigated this loss (Fig. 4C).

In addition, local injection of GW4869 had a considerable impact on IVDD, as shown by tissue fluorescence staining. The results showed that acupuncture-induced IVDD accelerated the senescence of NPCs, and when comparing the degeneration group to the Ctrl group, there were more P53- and P21-positive cells. Local injection of GW4869 reversed this effect. In the GW4869 group, P53 and P21 expression dropped by 41.8% and 40.3%, respectively, in comparison to the degeneration group (Fig. 4D). Additionally, the extracellular matrix catabolic index (MMP13) was higher in the degeneration group than in the Ctrl group, although the extracellular matrix anabolic index (Col-2) was lower in the former. In contrast, GW4869 restored the ECM metabolic balance (Fig. 4E). These findings provide compelling evidence that inhibition of M1-Exos may slow the development of IVDD.

### M1-Exos transport LCN2 to promote NPC senescence through the NF-κB signaling pathway

**Fig. 5:**
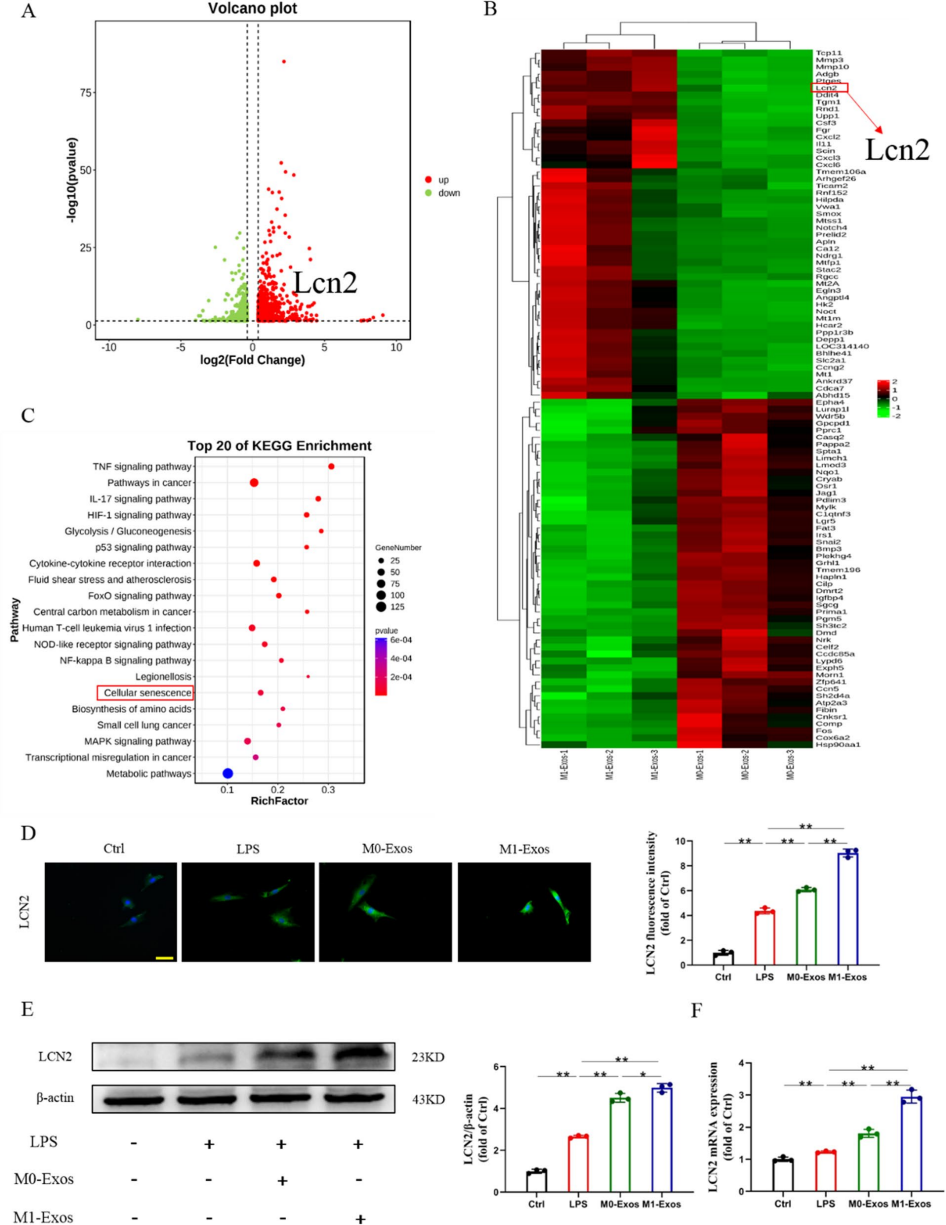
M1-Exos aggravates IVDD via LCN2. **A** Volcano plots of all differentially expressed genes (> 1.3 times) after M1-Exos and M0-Exos intervention with NPCs. **B** Heatmaps of all differentially expressed genes (> 1.3 times) after M1-Exos and M0-Exos intervention with NPCs. **C** The enriched KEGG pathways. **D** Representative fluorescent images of LCN2. Scale bars, 50 μm. **E** The expression and quantification of LCN2. **F** The mRNA levels of LCN2. ns: no significance, *P < 0.05, **P < 0.01

M0-Exos and M1-Exos were added to LPS-treated NPCs and cultured for 24 h. RNA sequencing (RNA-Seq) was performed to observe changes in the transcriptome. Our aim was to explore the undiscovered molecular mechanism by which M1-Exos regulate IVDD under inflammatory conditions. In the NPCs treated with M1-Exos, we identified 1572 genes in all (588 genes showed downregulation and 984 genes showed upregulation) whose expression differed markedly from that of the M0-Exos group (| fold change |> 1.3, *P* < 0.05, FDR < 0.05). The abundance of KEGG pathways suggests that aging may play a decisive role in this process. Excitingly, our analysis identified a gene, Lipocalin2 (Lcn2), that may play a key role in exacerbating NPC senescence in the context of M1-Exo intervention (Fig. 5A–C). The results were confirmed by immunofluorescence, western blot and RT‒PCR (Fig. 5D–F).

**Fig. 6:**
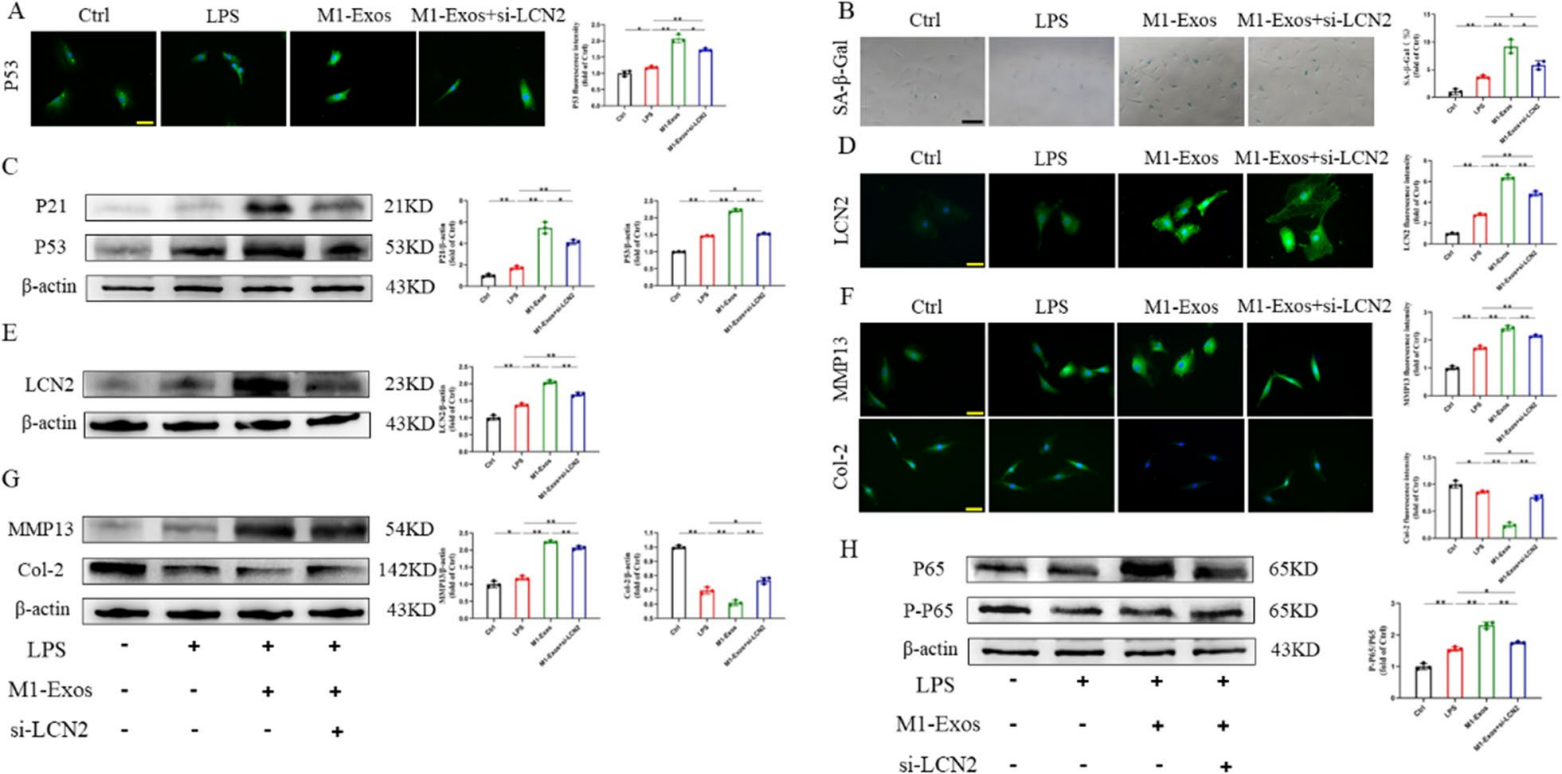
M1-Exos transport LCN2 promotes senescence of NPCs through NF-κB signaling pathway. **A** Representative fluorescence images of senescence markers after transfection with si-LCN2. Scale bars, 50 μm. **B** SA-β-gal staining and quantitative analysis results after transfection with si-LCN2. Scale bars, 100 μm. **C** The expression and quantification of senescence proteins after transfection with si-LCN2. **D** Representative fluorescent images of LCN2 after transfection with si-LCN2. Scale bars, 50 μm. **E** The expression and quantification of LCN2. **F** Representative fluorescence images of IVDD-specific markers after transfection with si-LCN2. Scale bars, 50 μm. **G** The expression and quantification of IVDD-specific proteins after transfection with si-LCN2. **H** The expression and quantification of NF-κB signaling pathway proteins after transfection with si-LCN2. ns: no significance, *P < 0.05, **P < 0.01

To confirm the function of LCN2 in regulating NPC senescence in M1-Exos, we used si-LCN2 to reduce LCN2 expression, which was validated by western blotting (Fig. S6). Immunofluorescence and Western blot results showed that M1-Exo injection aggravated cellular senescence events, including increased P53 and P21 expression. si-LCN2 decreased cellular senescence events (Fig. 6A, C). SA-β-gal staining showed a 36.4% decrease in positive cells in the M1-Exos + si-LCN2 group compared to the M1-Exos group (Fig. 6B). RT‒PCR results for age-related genes showed the same trend (Fig. S7A). In addition, ICC staining showed that, compared with that in the M1-Exos group, the fluorescence intensity of LCN2 in the M1-Exos + si-LCN2 group was much lower but higher than that in the LPS group (Fig. 6D). Western blot and RT‒PCR showed the same results (Fig. 6E, Fig. S7B). In addition, we also confirmed that si-LCN2 led to enhanced Col-2 expression and downregulated MMP13 expression, indicating that si-LCN2 could alleviate the aggravated ECM degradation of M1-Exos (Fig. 6F, G). The results were further confirmed by RT‒PCR (Fig. S7C). In summary, these data confirm that si-LCN2 can improve NPC senescence caused by M1-Exos and reduce IVDD.

There have been reports indicating that the NF-κB pathway facilitates the ageing process of cells [35]. Additionally, it is crucial to the development of IVDD [36]. An important function for the NF-κB pathway in this process is suggested by KEGG enrichment analysis. GSEA had similar results (Fig. S8A, B). Therefore, we investigated whether the NF-κB signaling pathway is involved in the effect of M1-Exos on LPS-induced NPC senescence. By using Western blot analysis, it was possible to determine that the NF-κB pathway was activated since the p-P65/P65 ratio in the LPS-treated NPCs was much greater than that in the Ctrl group. Furthermore, M1-Exo intervention further increased the p-P65/P65 ratio, while si-LCN2 treatment reversed this phenomenon (Fig. 6H). Thus, M1-Exos regulate the NF-κB pathway through LCN2 to accelerate NPC senescence and IVDD.

### Inhibition of LCN2 can delay the senescence of nucleus pulposus cells in vivo

**Fig. 7:**
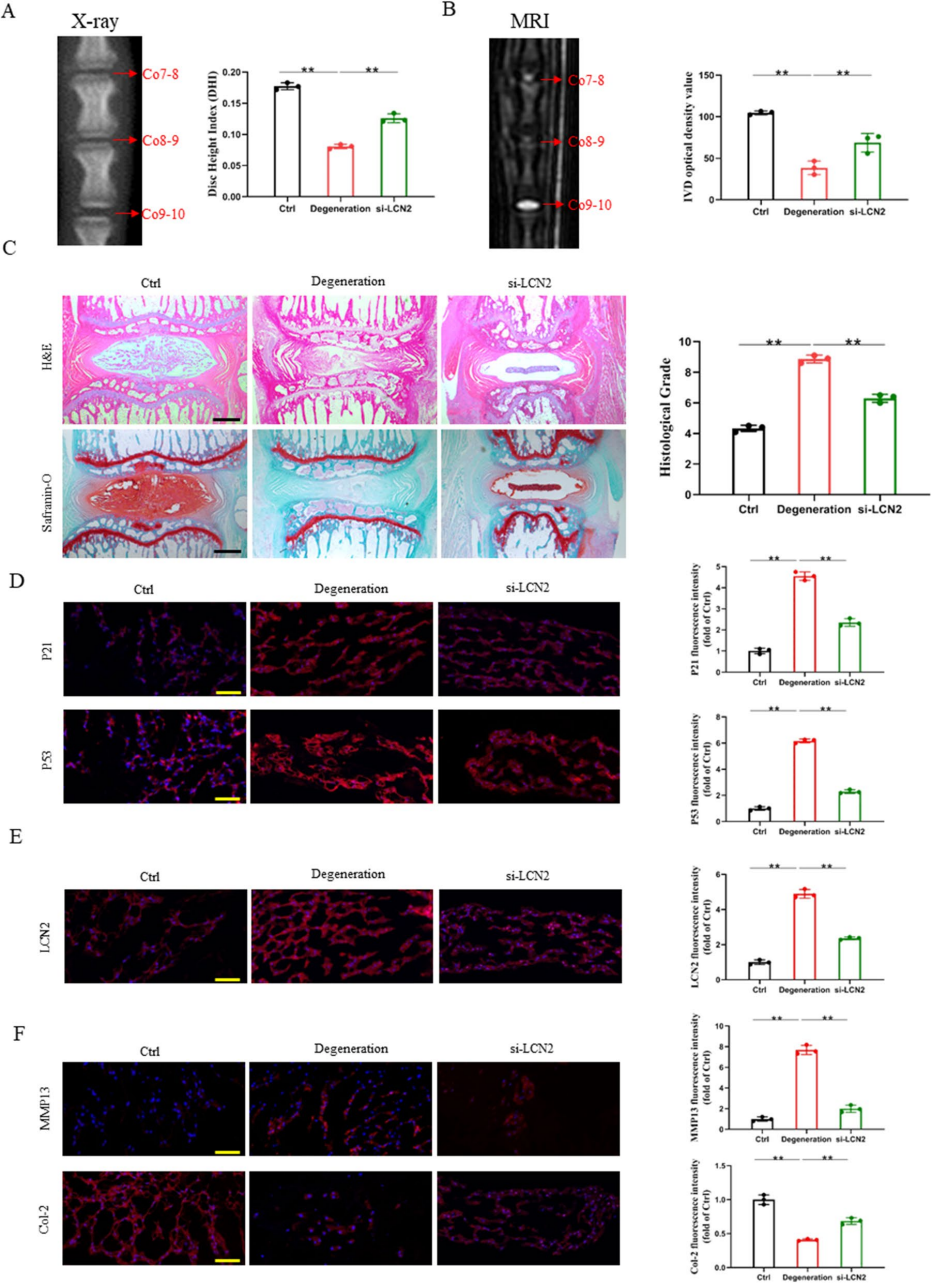
Inhibition of LCN2 can delay the senescence of nucleus pulposus cells in vivo. **A** X-ray examination of rat caudal at 1 week after surgery. **B** T2-weighted MRI scans of rat caudal at 1 week after surgery. **C** H&E and safranin-O staining of rat caudal IVD and histological grade. Scale bars, 1000 μm. **D** Representative fluorescent images of senescence markers after si-LCN2 intervention. Scale bars, 50 μm. **E** Representative fluorescent images of LCN2 after si-LCN2 intervention. Scale bars, 50 μm.** F** Representative fluorescent images of IVDD-specific markers after si-LCN2 intervention. Scale bars, 50 μm. ns: no significance, *P < 0.05, **P < 0.01

The effect of si-LCN2 was preliminarily evaluated by imaging. A week following the puncture, an MRI and X-ray were taken. On the seventh day after puncture, the rats in the puncture group showed a substantial drop in disc height, but the rats in the Ctrl group showed no change in disc height. The IVD puncture site in the degeneration group was far narrower than that in the Ctrl group, as illustrated in Fig. 7A. Remarkably, after si-LCN2 therapy, the decrease in disc height started to slow down. Compared with the Ctrl group, DHI decreased by 28.8% in the si-LCN2 group and 54.8% in the degeneration group at 7 days after puncture. According to the results of magnetic resonance imaging (MRI), the signal intensity of the degeneration group was significantly lower than that of the Ctrl group, indicating the loss of water in the disc of the degeneration group. When compared to the Ctrl group, the MRI signal of the punctured disc was much weaker; however, this was successfully mitigated by si-LCN2 therapy (Fig. 7B).

**Fig. 8:**
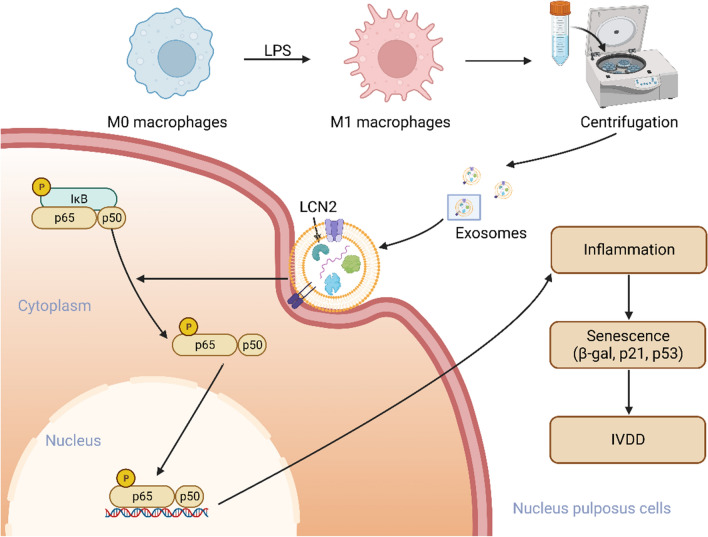
Schematic illustration of the mechanism related to LCN2 delivery by M1 macrophage-derived exosomes to promote nucleus pulposus cell senescence

Histological staining can clearly reveal the structure of the IVD. As shown in Fig. 8C, NP loss was caused by acupuncture 7 days after modelling. As a result, the NP volume is decreased, and the AF lamellar arrangement is bent and deformed in the degeneration group. Si-LCN2 therapy greatly preserved the whole structure of the NP and AF compared to the degeneration group. Upon needle puncture, vacuolar cells in the NP exhibit fibrocartilaginous alterations, as seen by Safranin-O staining. Si-LCN2 treatment prevented the loss of collagen and proteoglycans in the NP, thus easing their degeneration process (Fig. 7C). The si-LCN2 group had a substantially lower histology score than the degeneration group.

In contrast to the Ctrl group, which had considerably lower levels of P53 and P21 expression, the degeneration group had significantly greater levels of P53 and P21 expression, 6.2 and 4.6 times higher, respectively. Among them, compared with the degeneration group, the number of P53-positive cells in the si-LCN2 group decreased by 58.5%, and the number of P21-positive cells decreased by 48.4% (Fig. 7D). LCN2 expression was detected in the NP area of rats, which allowed us to look into the precise locations where si-LCN2 alleviates IVDD. As shown in Fig. 7E, the expression level of LCN2 in the NP in the degeneration group was significantly higher than that in the other groups and was reversed after si-LCN2 treatment. To assess the level of IVDD in various groups, tissue fluorescence staining of NP using Col-2 and MMP13 was carried out. Col-2 expression was strong in the Ctrl group and low in the degeneration group, indicating ECM degradation and remodelling in the degeneration group. The expression level of the si-LCN2 group was between that of the two groups. The number of Col-2-positive regions in the NP in the Ctrl group and the si-LCN2 group was 2.5 times and 1.7 times that in the degeneration group, respectively. However, the expression of MMP13 was opposite to that of Col-2 (Fig. 7F). These findings imply that exogenous si-LCN2 treatment may considerably slow the advancement of IVDD.

## Discussion

It is generally believed that IVDD is more common in old age. This seems logical because with age, tissue degeneration increases and the ability to heal decreases. Surprisingly, in our study, we found that the proportion of young people with LDH increased. From the perspective of MRI, in young patients with LDH, in addition to the degeneration of the segment-visible HIZ (a kind of injury signal), the rest of the segmental disc signal is still good. These are the results of the changes in the way young people live in today's society. It has been reported that obesity, long-term abnormal stress, and trauma are important reasons for LDH in young patients. Proinflammatory adipokines and systemic inflammation characteristic of obese individuals have been reported as promoters of IVDD [37]. In addition, exercise-related injuries and a history of trauma, such as traffic accidents, are also causes of IVDD in young people [38]. All these factors are accompanied by inflammation and are closely related to the imbalance of the inflammatory microenvironment in IVD. The inflammatory microenvironment leads to the occurrence of IVDD, which leads to tissue atrophy, fibrosis, and phenotypic changes in NPCs [39]. At the same time, IVDD causes the inflammatory microenvironment to emerge, which is exacerbated or created by highly expressed inflammatory mediators (TNF-α, ILs, etc.) in the tissues [40]. In addition, IVD within the inflammatory microenvironment can recruit more exogenous inflammatory cells, which exacerbates the inflammatory severity of IVDD [41]. In our study, we found significant elevations in IL-6 and IL-1β in young LDH patients. One of the main inflammatory factors in the IVDD microenvironment is IL-1β, which can increase the production of additional inflammatory mediators, such as IL-6, IL-8, IL-17, PGE2, chemokines and the NLRP3 inflammasome, to form a sustained inflammatory microenvironment [42]. T cells, macrophages, and NPCs release IL-6, a multifunctional cytokine that may accelerate the ECM destruction of NPCs [43]. Primary producers of IL-17 are the helper T-cell 17 subgroup of CD4 + T cells. Research has revealed that IL-17A is markedly upregulated in human degenerative IVD tissues, affects the metabolic balance of the extracellular matrix through various pathways, including NF-κB and JAK/STAT, and contributes to the inflammatory environment that advances IVDD [44]. Interestingly, there was no significant difference in the amount of TNF-α expressed in our results, which might be due to the small sample size.

Macrophages are significant immune cells that have the ability to interact with osteoclasts, regulate bone metabolism via the secretion of various cytokines and are crucial for the growth and maintenance of bone homeostasis [45]. In general, LPS or IFN-γ may polarize macrophages into M1 macrophages, which stimulates the generation of inflammation. IL-4 or IL-13 polarizes macrophages into M2 types that aid in tissue repair and inflammation resolution. The fate of tissues inside the inflammatory environment is dictated by the polarization balance of M1/M2 macrophages. Continuous polarization in the direction of M1 will cause the destruction of the inflammatory microenvironment, which will promote the large production of proinflammatory cytokines, thus accelerating the occurrence of inflammation, which has been reflected in a variety of inflammatory diseases, such as atherosclerosis, obesity, tumors, asthma and sepsis [46]. According to our single-cell sequencing results, macrophages are present in the degenerative IVD, which is consistent with previous findings [11]. Tu et al. isolated NP tissues from 8 IVDD patients with different degrees of degeneration for scRNA-seq and found that macrophages with CD163 as a marker were present in NP tissues [47]. In addition, the IVDD cell-to-cell interaction network found that macrophages interact most with other cell types. Immunomodulation-related cytokines produced by macrophages, such as TNF-α and IL-6, can bind to NPC receptors. Macrophages express growth factors, including TGFB, PDGF and OSM, and exhibit proliferative effects on NPCs. Next, we reclassified the macrophages in the IVD and found that compared with the normal group, there were a large number of M1-dominated macrophages in the degeneration group, suggesting that inflammation plays a key role in IVDD. Li et al. defined five different subtypes of mononuclear/macrophages during IVDD [48]. “Oxidative stress (OS)-associated macrophages” with similar functions to M1 macrophages are dominant in advanced degenerative IVD tissues and are enriched in functions such as “acute inflammatory response”, “ROS metabolic process” and “oxidative stress response”. In addition, highly activated “OS-associated macrophages” were mainly enriched in proinflammatory mediators and secreted large amounts of IL1A, IL1B, CXCL8 and TNF-α. These findings suggest that M1 macrophages undergo the activation of an inflammatory response during IVDD, forming a proinflammatory microenvironment within the IVD. Therefore, targeting macrophages for polarization and tilting their phenotype to restore balance to the inflammatory microenvironment may hold great promise for treating IVDD.

Interestingly, in our study, we found by RNA-Seq that targeting LCN2 derived from M1 macrophage exosomes can affect NPC senescence and regulate the IVDD process. Lipocalin2 (LCN2), also known as neutrophil gelatinase-associated lipid carrier protein (NGAL), is a secreted protein that is a member of the lipocalin superfamily and has multiple functions in immune response and cell signalling [49]. Currently, LCN2 is considered a potential molecular therapeutic target for IVDD therapy based on the gene expression profile of disc cells [50]. In our experiment, inhibition of LCN2 mitigated NPC senescence and IVDD induced by M1-Exos. This may be attributed to LCN2's ability to bind to MMP9 to enhance matrix degradation [51]. As proposed by Kao et al., the dimerization of LCN2 with proMMP9 enhances activation of the enzyme by plasma kallikrein and protects the enzyme from degradation, thereby maintaining the activity of the MMP9 protein [52]. There are processes that may enhance IVDD. As an important regulatory target, LCN2 is intimately associated with many processes, such as ferroptosis, autophagy [53], and apoptosis [54]. Similarly, LCN2 was found to affect NPC senescence in our study. Jiang et al. demonstrated that genetic manipulation of LCN2 gene expression can regulate astrocyte senescence and control the progression of Parkinson’s disease [55]. In summary, we speculated that LCN2 could affect the degeneration of NPCs through senescence.

An essential nuclear transcription factor, nuclear factor-κB (NF-κB), regulates innate immunity, acquired immunity, the inflammatory response, and the development of tumours [56]. The increase in matrix-degrading enzymes and proinflammatory cytokines results from its activation in IVDD. Furthermore, as a consequence of decreased proteoglycan and type II collagen breakdown, suppression of the NF-κB pathway prevents the overexpression of MMPs and ADAMTS [57]. The NF-κB pathway has also been implicated in NPC senescence. According to Li et al., by controlling the ROS/NF-κB pathway, 17beta-estradiol prevented TNF-α-induced early senescence of NPCs [58]. On this basis, we further investigated whether LCN2 is related to the NF-κB pathway. According to our findings, the M1-Exos group had considerably higher levels of NF-κB activation, while the p-p65/p65 ratio was significantly reduced after the injection of si-LCN2. This implies that the activation of the NF-κB pathway in IVDD may be inhibited by inhibiting LCN2. The heterodimer of p50 and p65 that makes up the most prevalent form of NF-κB is often found in the cytoplasm and does not bind to the IκB protein or have transcriptional activity. Our results show that LCN2 significantly reduces the nuclear translocation of p65 by inhibiting NF-κB promoter activity, thereby inhibiting the aging of NPCs. This is consistent with the results of Feng et al. [59]. However, Huang et al. showed that there is a regulatory factor RPS3 between LCN2 and NF-κB. When LCN2 expression is reduced, the binding of RPS3 to NF-κB is affected, which reduces the activation of NF-κB [60]. This suggests that LCN2 may indirectly regulate NF-κB activity, but its specific mechanism in the effect of M1-exos on IVDD needs to be further explored.

IVDs are complex fibrocartilage joints, often referred to as the largest vaseless structure in the body [61]. Under normal circumstances, the IVD lacks a blood supply, and exogenous inflammatory cytokines have difficulty reaching the interior of the IVD [62]. In addition, degraded NPCs can release many proinflammatory factors, recruit exogenous macrophages, and further release proinflammatory factors, leading to an inflammatory cascade and aggravating IVDD [63]. Therefore, how the substances secreted by exogenous macrophages reach the IVD has become a difficult problem. The main role of exosomes is thought to be as a medium of intercellular communication, which can deliver proteins, lipids and nucleic acids (mRNA and miRNA) to the recipient cell, effectively altering the biological response of the recipient cell [64]. The cargo contents of exosomes are specific to the origin cell and can transmit parental cell signals to neighboring cells or target cells without direct cell-to-cell contact [65]. In addition, exosomes contribute to the maintenance of homeostasis and healing of diseases through tissue-tissue and cell-to-cell communication. The positive effects of exosomes have been demonstrated in a variety of diseases [66–68]. Similarly, an essential part in IVDD is played by exosomes. Exosomes from mesenchymal stem cells regulate endoplasmic reticulum stress and prevent nucleus pulposus cell death [69]. By releasing exosomes to NPCs and activating the PI3K/AKT/autophagy pathway, cartilaginous endplate stem cells prevent IVDD [70]. Similarly, in our study, exosomes from M1 macrophages acted as carriers to deliver LCN2 to NPCs, further worsening IVDD. However, NPC senescence was alleviated after treatment with the exosome inhibitor GW4869, which inspired us that exosomes could be a novel therapy and open the way for the treatment of IVDD.

However, there are still some shortcomings in this study. First, the influence of M1 macrophages on IVDD was the main focus of this article; M2 macrophages were not studied. Second, we only studied the association between M1-Exos and cellular senescence in NPCs during IVDD. Future studies will focus on determining whether a similar link exists between M1-Exos and annulus fibrosus cells and endplate chondrocytes. Third, exosomes, as a delivery medium, can deliver miRNA in addition to mRNA, which is a future research direction in IVDD. Finally, in addition to cellular senescence, there are other mechanisms of action of LCN2, such as ferroptosis, that play a role in IVDD.

## Conclusion

Our findings demonstrate that M1 macrophage-derived exosomes may transport LCN2, which aggravates NPC senescence and worsens IVDD. This activation of the NF-κB signaling pathway is achieved by exosome delivery. This study sheds new light on the pathogenesis of young IVDD patients and has the potential to provide personalized treatment options.

### Supplementary Information

Additional file 1.

## Data Availability

The datasets generated and/or analysed during the current study are not publicly available but are available from the corresponding author upon reasonable request.

## Abbreviations

IVDD: Intervertebral disc degeneration
LBP: Low back pain
NPCs: Nucleus pulposus cells
LPS: Lipopolysaccharide
M1-Exos: M1 macrophage-derived exosomes
M0-Exos: M0 macrophage-derived exosomes
Col-2: Type II collage
MMP13: Matrix metalloproteinase 13
ECM: Extracellular matrix
IVDs: Intervertebral discs
LDH: Lumbar disc herniation
HIZ: High-intensity zone
LCN2: Lipocalin2
NGAL: Neutrophil gelatinase-associated lipid carrier protein
NF-κB: Nuclear factor-κB
