# Supplementary material for: M1 macrophage-derived exosomes promote intervertebral disc degeneration by enhancing nucleus pulposus cell senescence through LCN2/NF-κB signaling axis
Source: J Nanobiotechnology. 2024 May 31;22:301. doi: 10.1186/s12951-024-02556-8 (PMC11140985; doi:10.1186/s12951-024-02556-8)
Supplement: Supplementary file 1 — Additional file 1. [file 12951_2024_2556_MOESM1_ESM.docx]

**Table S1. Chemicals and Antibodies**

| Name | Source | Identifier |
| --- | --- | --- |
| Dimethyl sulfoxide  (DMSO) | Sigma-Aldrich | CAS#67-68-5 |
| Lipopolysaccharide  (LPS) | Sigma-Aldrich | EINECS#297-473-0 |
| GW4869 | Sigma-Aldrich | CAS#[6823-69-4](https://www.sigmaaldrich.cn/CN/zh/search/6823-69-4?focus=products&page=1&perpage=30&sort=relevance&term=6823-69-4&type=cas_number) |
| iNOS antibody | Abclonal | A3774 |
|  |  | Dilution: WB 1:1000 |
| CD86 antibody | Abclonal | A16805 |
|  |  | Dilution: WB 1:500; IHC 1:100 |
| CD68 antibody | Abcam | Ab201340 |
|  |  | Dilution: IHC 1:100 |
| TSG101 antibody | ZCIBIO | ZC-216049 |
|  |  | Dilution: WB 1:1000 |
| CD63 antibody | ZCIBIO | ZC-211909 |
|  |  | Dilution: WB 1:300 |
| Col-2 antibody | Abcam | Ab34712 |
|  |  | Dilution: WB 1:5000; IHC 1:200 |
| MMP13 antibody | Abcam | Ab39012, |
|  |  | Dilution: WB 1:5000, IHC 1:200 |
| CDKN1A/p21CIP1 antibody | Abclonal | A1483 |
|  |  | Dilution: WB 1:1000; IHC 1:100 |
| p53 antibody | Abclonal | A0263 |
|  |  | Dilution: WB 1:1000; IHC 1:100 |
| LCN2 antibody | Proteintech | 26991-1-AP |
|  |  | Dilution: WB 1:1000; IHC 1:100 |
| NF-kB p65 antibody | Abclonal | A19653 |
|  |  | Dilution: WB 1:1000 |
| Phospho-NF-kB p65 (Ser536) antibody | Affinity | Af2006 |
|  |  | Dilution: WB 1:1000 |
| β-actin antibody | Abcam | Ab6276 |
|  |  | Dilution: WB 1:5000 |
| Phalloidin-iFluor 488 | Abcam | Ab176753 |
|  |  | Dilution: IHC 1:1000 |
| Alexa Fluor@647 | Abcam | Ab150135 and 150115 |
|  |  | Dilution: IHC 1:1000 |
| Goat Anti-Rabbit IgG  secondary antibody | Abcam | Ab205718 |
|  |  | Dilution: WB 1:5000 |
| DAPI | Abcam | Ab104139 |
|  |  | Dilution: IHC 1:20 |

**Table S2. Primers based on the rat genome used for RT–PCR**

| **Gene** | **Forward primers** | **Reverse primers** |
| --- | --- | --- |
| **GAPDH** | GCAAGTTCAACGGCACAG | CGCCAGTAGACTCCACGAC |
| **Col-2** | GAGTGGAAGAGCGGAGACTACTG | CTCCATGTTGCAGAA  GACTTTCA |
| **MMP13** | TCCATCCCGAGACCTCATGT | AGCATCATCATAACTCCACACG |
| **TP53** | CACCTCCACACCTCCACCTG | GCCGTCACCATCAGAGCAAC |
| **CDKN1A** | GACCTAAGCGTACCGTCCAGAG | GAGAGCAGCAGATCACCAGATTAAC |
| **LCN2** | CCGACACTGACTACGACCAG | AATGCATTGGTCGGTGGGAA |

Sig 1


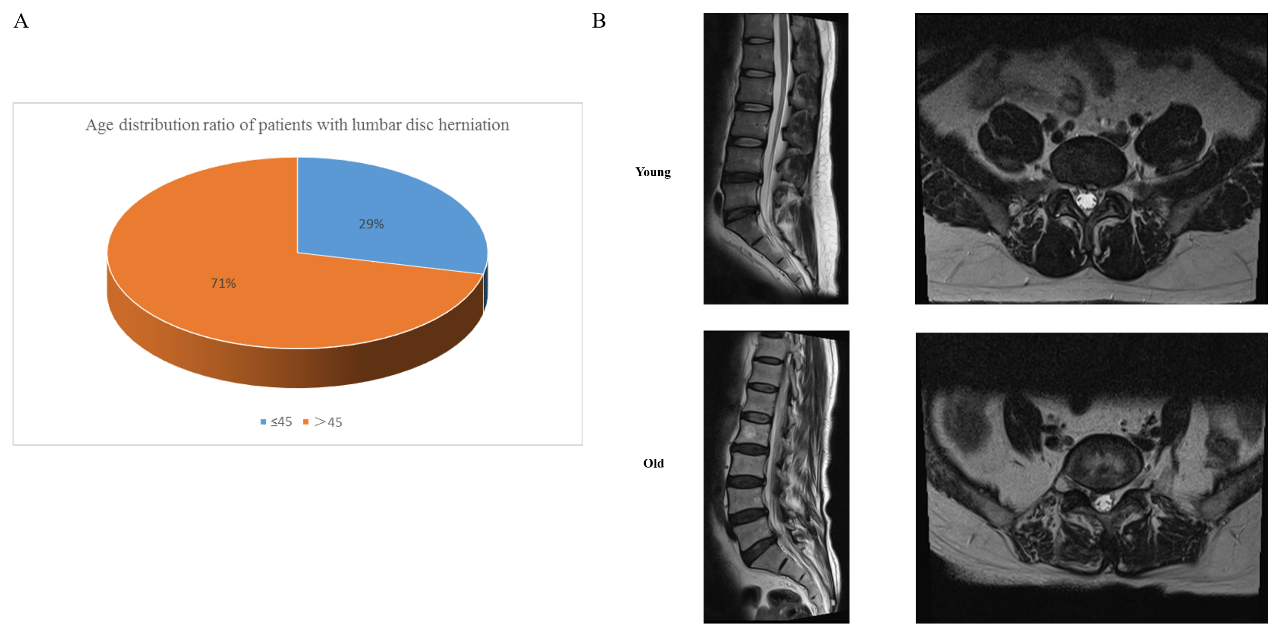


**Sig 1 A** The age proportion of 727 LDH patients in the First Affiliated Hospital of Soochow University from September 2022 to September 2023 (with 45 years as the boundary). **B** MRI results in young and elderly LDH patients.

Sig 2


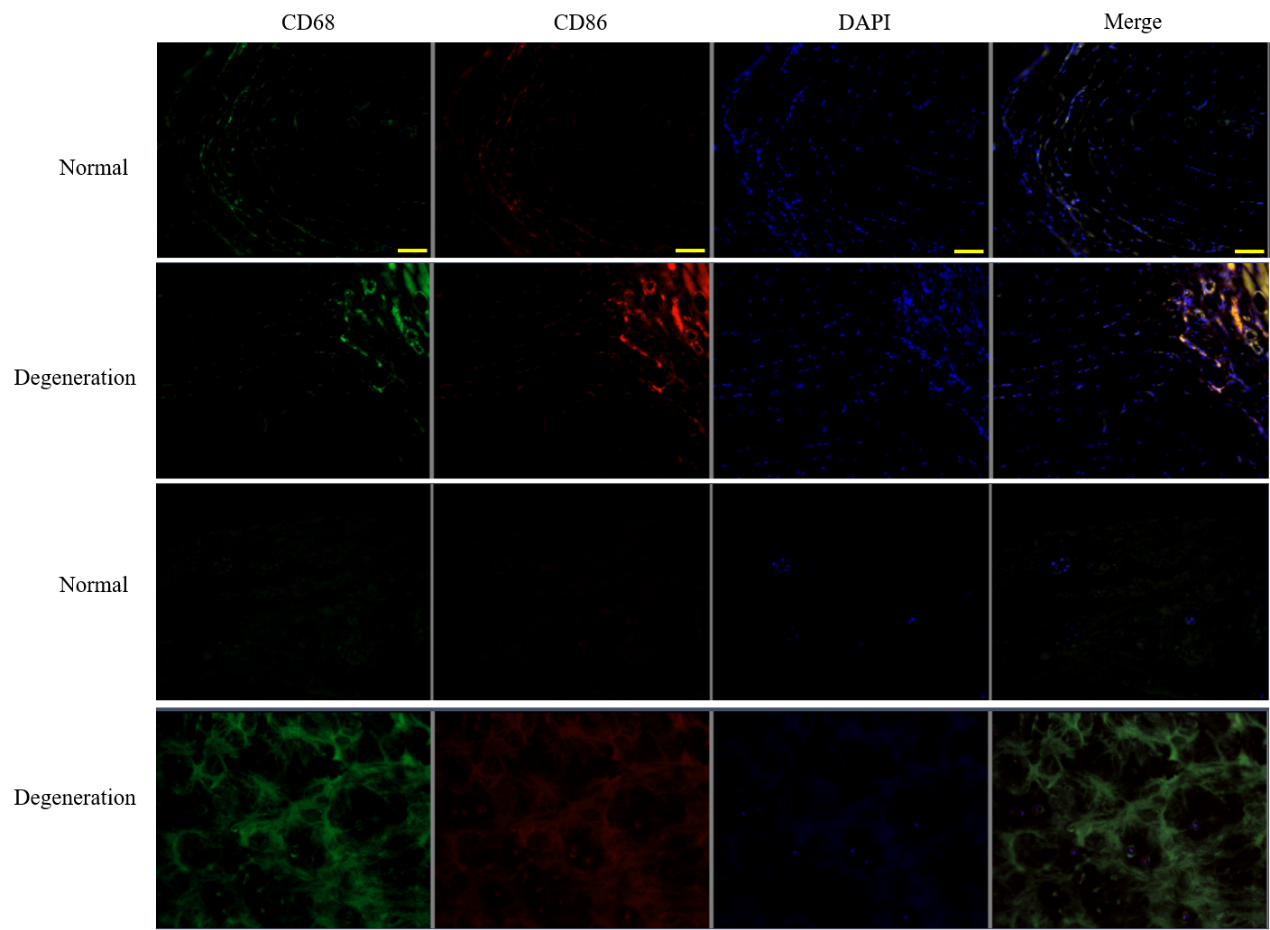


**Sig 2** The co-immunofluorescence of CD68 and CD86 in rat and human tissues. Scale bars, 100 μm.

Sig 3


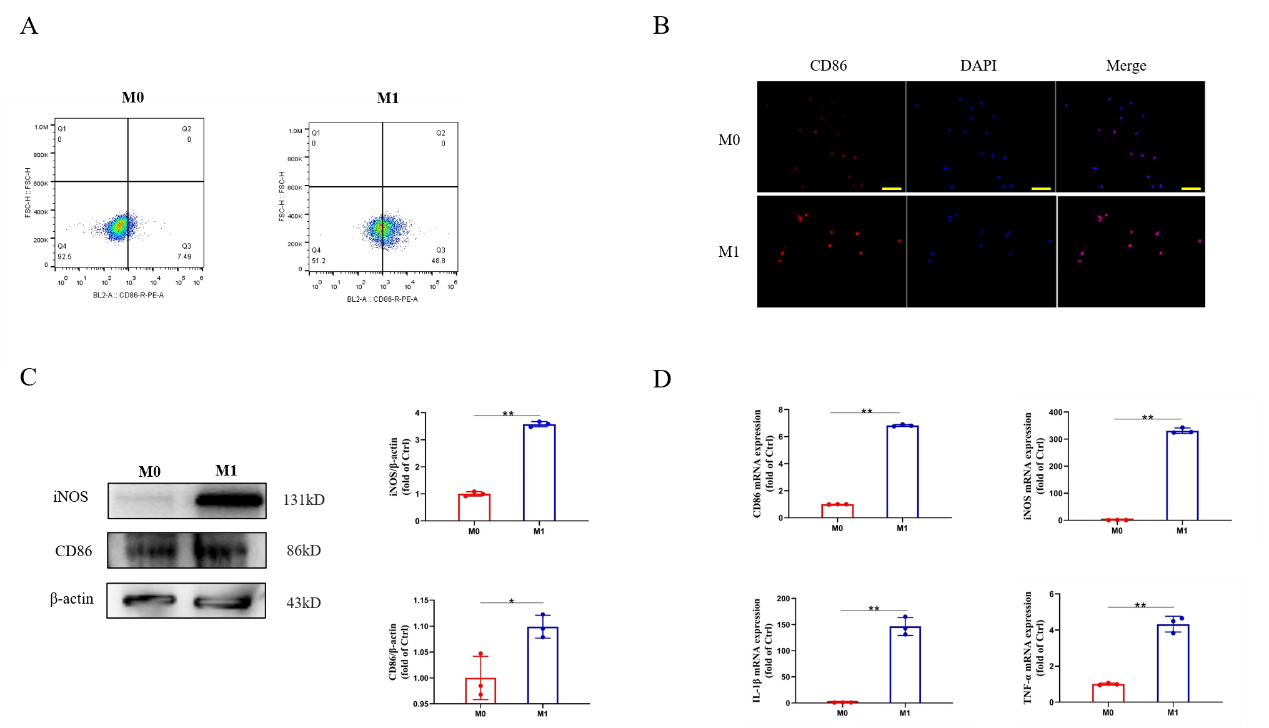


**Sig 3** Macrophages were polarized to M1 phenotype by LPS intervention. **A** Flow cytometry results. **B** Representative fluorescent images of CD86. Scale bars, 100 μm. **C** The expression and quantification of M1 macrophage marker proteins. **D** The expression of macrophage marker genes. ns: no significance, *p < 0.05, **p < 0.01.

Sig 4


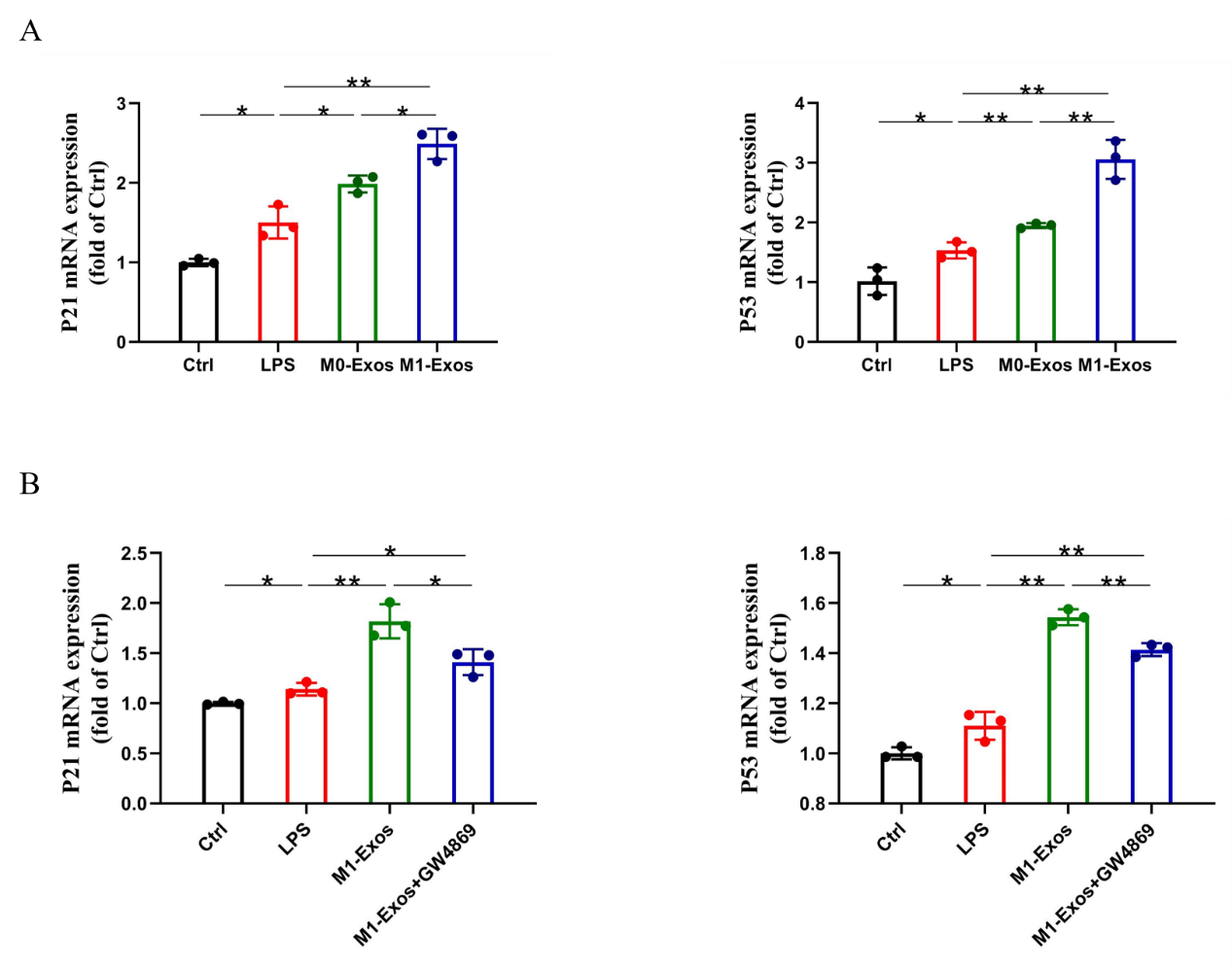


**Sig 4 A** The expression of senescence genes after addition of M1-Exos. **B** The expression of senescence genes after GW4869 intervention. ns: no significance, *p < 0.05, **p < 0.01.

Sig 5


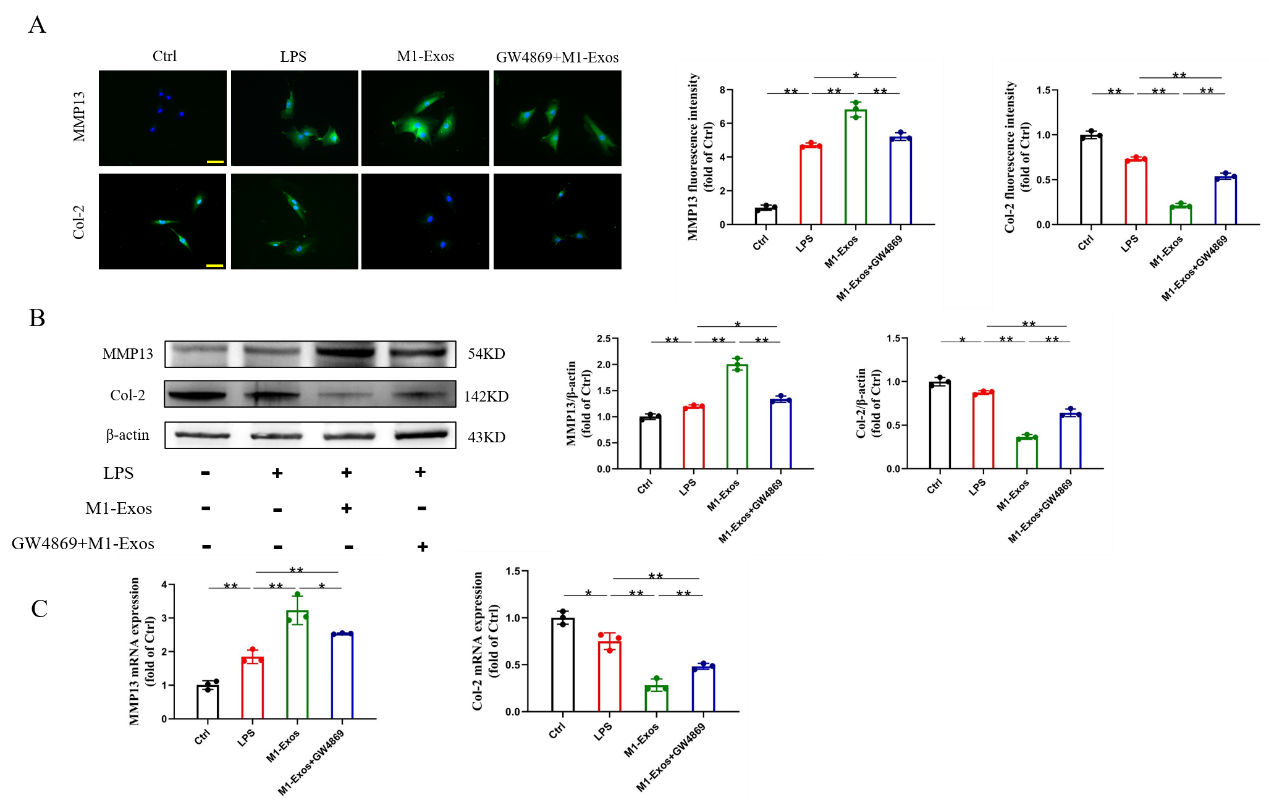


**Sig 5 A** Representative fluorescent images of IVDD-specific markers after GW4869 intervention. Scale bars, 50 μm. **B** The expression and quantification of senescence proteins after GW4869 intervention. **C** The expression of senescence genes after GW4869 intervention. ns: no significance, *p < 0.05, **p < 0.01.

Sig 6


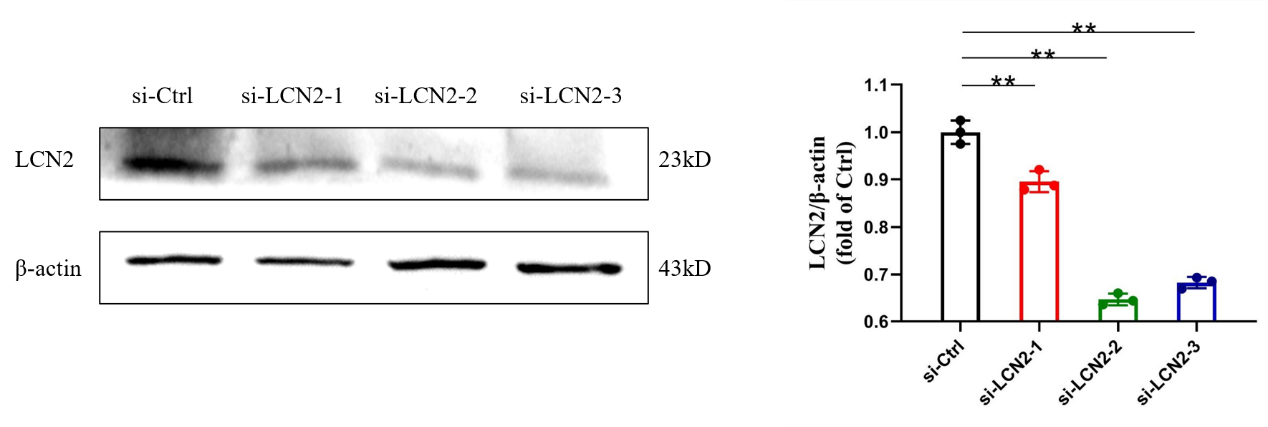


**Sig 6** The silencing effect of LCN2 si-RNA confirmed by western blot and quantification analysis. ns: no significance, *p < 0.05, **p < 0.01.

Sig 7


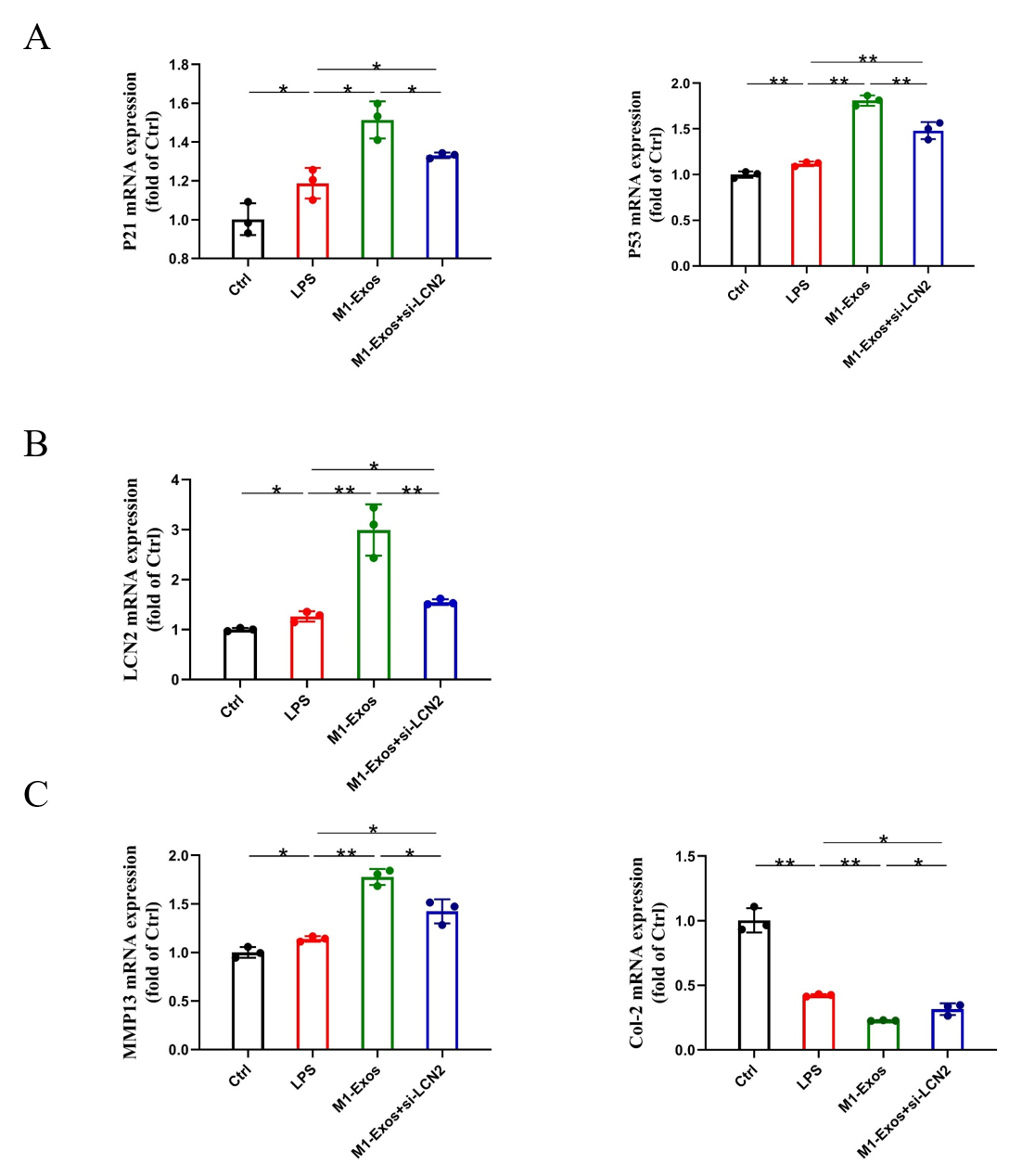


**Sig 7 A** The expression of senescence genes after transfection with si-LCN2. **B** The expression of LCN2 after transfection with si-LCN2. **C** The expression of IVDD-specific genes after after transfection with si-LCN2. ns: no significance, *p < 0.05, **p < 0.01.

Sig 8


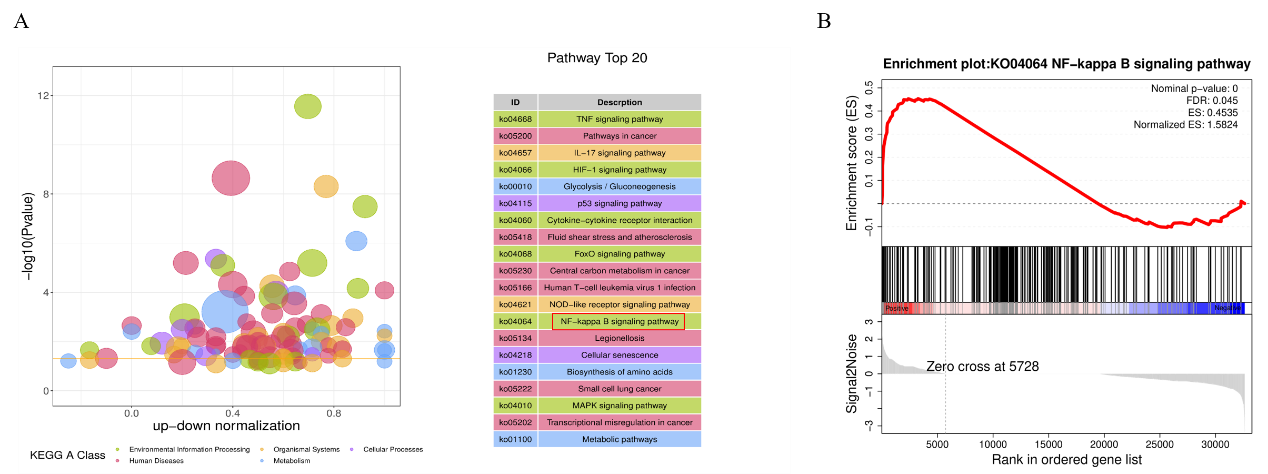


**Sig 8 A** The enriched KEGG pathways. **B** Gene Set Enrichment Analysis (GSEA) of NF-κB signaling pathway.
